# Norepinephrine‐CREB1‐miR‐373 axis promotes progression of colon cancer

**DOI:** 10.1002/1878-0261.12657

**Published:** 2020-03-13

**Authors:** Jia Han, Qiuyu Jiang, Ruili Ma, Huahua Zhang, Dongdong Tong, Kaijie Tang, Xiaofei Wang, Lei Ni, Jiyu Miao, Baojun Duan, Yang Yang, Yanke Chen, Fei Wu, Jiming Han, Mengchang Wang, Ni Hou, Chen Huang

**Affiliations:** ^1^ Department of Cell Biology and Genetics School of Basic Medical Sciences Xi’an Jiaotong University Health Science Center China; ^2^ School of Basic Medical Science Xi’an Medical University China; ^3^ Medical College Yan'an University China; ^4^ Department of Medical Oncology The Third Affiliated Hospital to Xi'an Jiaotong University China; ^5^ Department of Health Toxicology and Hygiene Inspection School of Public Health Xi'an Jiaotong University Health Science Center China; ^6^ Department of Hematology The First Hospital Affiliated to Xi'an Jiaotong University China; ^7^ Key Laboratory of Environment and Genes Related to Diseases Ministry of Education of China Xi’an China; ^8^ Key Laboratory of Shaanxi Province for Craniofacial Precision Medicine Research College of Stomatology Xi'an Jiaotong University China

**Keywords:** colorectal cancer, CREB1, miR‐373, NE, TIMP2

## Abstract

The adrenergic system contributes to the stress‐induced onset and progression of cancer. Adrenergic fibers are the primary source of norepinephrine (NE). The underlying mechanisms involved in NE‐induced colon cancer remain to be understood. In this study, we describe the function and regulatory network of NE in the progression of colon cancer. We demonstrate that NE‐induced phosphorylation of cAMP response element‐binding protein 1 (CREB1) promotes proliferation, migration, and invasion of human colon cancer cells. The downstream effector of NE, CREB1, bound to the promoter of miR‐373 and transcriptionally activated its expression. miR‐373 expression was shown to be necessary for NE‐induced cell proliferation, invasion, and tumor growth. We confirmed that proliferation and invasion of colon cancer cells are regulated *in vitro* and *in vivo* by miR‐373 through targeting of the tumor suppressors TIMP2 and APC. Our data suggest that NE promotes colon cancer cell proliferation and metastasis by activating the CREB1–miR‐373 axis. The study of this novel signaling axis may provide mechanistic insights into the neural regulation of colon cancer and help in the design of future clinical studies on stress biology in colorectal cancer.

## Graphical abstract

We propose a NE–CREB1–miR‐373 signaling axis in human colon cancer cells. NE promotes the progression of colon cancer via CREB1 activity‐dependent transcription, enhancing the expression of oncogenic miR‐373 and reducing the expression of the miR‐373 targets APC and TIMP2. This novel NE‐CREB1‐miR‐373 signaling axis may potentially serve as a new therapeutic target for colorectal cancer.

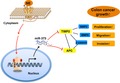

**Abbreviations**
APC: adenomatous polyposis coli
CRC: colorectal cancer
CREB1: cAMP response element‐binding protein 1
NE: norepinephrine
qRT‐PCR: quantitative real‐time polymerase chain reaction
TCGA: The Cancer Genome Atlas
TH: tyrosine hydroxylase
TIMP2: tissue inhibitor of metalloproteinases 2

## 1 Introduction

Organisms continuously encounter a variety of stresses that cause maladaptation and may even result in cancer. Increasing evidence indicates the importance of stress in cancer progression. Stress mediators such as neuroendocrine hormones enhance cancer pathogenesis by inhibiting antitumor immune responses; recent studies suggest that the peripheral nerves in tumor‐nervous connections (TNCs) modulate the physiological behavior of cancer cells and affect cancer progression (Amit *et al.*, 2016; Chida *et al.*, 2008; Faulkner *et al.*, 2019; Jobling *et al.*, 2015; Mauffrey *et al.*, 2019; Quaegebeur *et al.*, 2011; Reiche *et al.*, 2004; Thaker *et al.*, 2006). Colorectal cancer (CRC) is a commonly occurring cancer that is densely innervated by autonomic fibers and results in shortened patient survival (Liebl *et al.*, 2013). TNCs consist of sympathetic and parasympathetic nervous system (Arese *et al.*, 2018; Faulkner *et al.*, 2019; Jobling *et al.*, 2015). Activation of the adrenergic system significantly affects stress‐induced cancer progression (Magnon *et al.*, 2013). Adrenergic fibers are the primary source of norepinephrine (NE). Although it is known that physiological changes are induced by the adrenergic system, the cellular targets and molecular mechanisms involved in the neural regulation of CRC remain to be investigated; the results of such studies may provide a basis for novel therapeutic interventions (Coelho *et al.*, 2017).

Sympathetic nerve fibers deliver adrenergic signals that are recognized by β‐adrenergic receptors (β‐ARs) present in the tumor microenvironment (Cole *et al.*, 2015). NE is the principal messenger released by central noradrenergic and peripheral sympathetic nerve fibers (Tang *et al.*, 2013). NE regulates cell proliferation, survival, and tumor progression by activating the β‐AR‐cyclic AMP (cAMP)–protein kinase A (PKA) pathway in various cancer cells (Cole and Sood, 2012). The transcription factor cAMP response element‐binding protein (CREB), which acts downstream of PKA, mediates stress‐induced changes in learning and memory (Peng *et al.*, 2015). Moreover, CREB stimulates the expression of miR‐373 and mediates the growth of zinc transporter 4‐induced pancreatic cancer (Zhang *et al.*, 2013). However, little is known about the role of CREB in stress‐induced progression of CRC.

MicroRNAs (miRs) are involved in the regulation of gene expression and may potentially serve as new therapeutics in cancer (Awasthi *et al.*, 2018). miR‐373 plays a variety of roles in tumorigenesis and tumor progression. It targets the epidermal growth factor receptor and functions as a tumor suppressor in glioblastomas; it also targets Dickkopf‐1 in the Wnt/β‐catenin pathway, thereby functioning as an oncogene in tongue squamous cell, testicular germ cell, esophageal squamous cell, and prostate cancer (Jing *et al.*, 2017; Voorhoeve *et al.*, 2006; Wei *et al.*, 2015; Weng *et al.*, 2017). In this study, using loss‐/gain‐of‐function, ChIP, and luciferase reporter assays, we reveal the importance of the NE–CREB1–miR‐373 axis and the oncogenic role of miR‐373 in colon cancer. Knowledge of this novel signaling axis may provide mechanistic insights into the neural regulation of CRC and help in the design of future clinical studies on stress biology in cancer.

## 2 Materials and methods

### 2.1 Reagents for cell culture and transfection

HCT116 and RKO cells were purchased from the American Type Culture Collection (Manassas, VA, USA) and cultured and transfected as described previously (Li *et al.*, 2010). The interference oligonucleotides used in this study were synthesized by GenePharma (Shanghai, China) and are listed in Table S1. The plasmids used for overexpression (CREB1, TIMP2) and their respective controls (CON, Vehicle) were purchased from GenePharma. The precursor of hsa‐miR‐373 was subcloned into pcDNA6.2 (Invitrogen, Carlsbad, CA, USA) and labeled as miR‐373; the blank vector used as a control was labeled as miR‐Ctrl. NE and propranolol were purchased from Sigma‐Aldrich and used at a molarity of 10 μm.

### 2.2 MTT assay

Cells were seeded in 96‐well plates and cultured for 24, 48, or 72 h before the addition of MTT; the absorbance of each well was then measured spectrophotometrically at 490 nm using a FLUOstar OPTIMA spectrophotometer (BMG Labtech, Ortenberg, Germany).

Each experiment was independently performed three times.

### 2.3 Colony‐forming assay

Colon cancer cells were seeded in 24‐well plates at a density of 5 × 10^2^ cells per well. After 1 week, the cells were stained with 0.1% (w/v) crystal violet and photographed.

Each experiment was independently performed three times.

### 2.4 Wound healing assay

Cells were seeded in 6‐well plates and incubated until they were 90% confluent. A straight scratch was then made across the base of the well. Images of the cells were captured at 40× magnification (Nikon, Tokyo, Japan) at 0, 24, and 48 h and used to determine cell migration. The width of the wound was measured at 0, 24, and 48 h using imagej (National Institute of Mental Health, Bethesda, MD, USA), and the results were used to quantify the rate of cell migration. Each experiment was independently performed three times.

### 2.5 Transwell assay

Cell suspensions (100 μL) comprising 1 × 10^5^ cells were seeded into the upper chambers of transwell plates lacking Matrigel for the migration assay or containing 30 μL Matrigel (BD Biosciences, San Jose, CA, USA) for the invasion assay. The upper chamber contained medium without fetal bovine serum, and the lower chamber was filled with 600 μL of medium containing 10% fetal bovine serum. The cells were cultured under normal conditions for 24 h for the migration and invasion assays. Subsequently, the incased migrating cells (at the bottom of the upper chamber) were stained with 0.1% (w/v) crystal violet and imaged at 100× magnification. Each experiment was independently performed three times.

### 2.6 Western blotting

Proteins were isolated, electrophoresed, and transferred to polyvinylidene fluoride membranes (Millipore, Billerica, MA USA). The transferred membranes were blocked and incubated sequentially with primary (1 : 1000 dilution) and secondary antibodies (1 : 5000 dilution, Jackson ImmunoResearch Laboratories, Inc., West Grove, PA, USA). The immunoreactive bands were visualized using enhanced chemiluminescence. The following primary antibodies were used: phospho‐CREB (CST 9198); CREB (CST 9197); tissue inhibitor of metalloproteinases 2 (TIMP2; CST 5738); adenomatous polyposis coli (APC; Proteintech 19782‐1‐AP); β‐catenin (Proteintech 51067‐2‐AP); and tyrosine hydroxylase (TH; Proteintech 66334‐1‐Ig). All experiments were independently performed three times.

### 2.7 Immunofluorescence assay

Cells on slides were fixed using 4% paraformaldehyde and permeabilized with 0.05% Triton X‐100. They were then incubated with primary antibodies (phospho‐CREB, 1 : 500 dilution; APC, 1 : 250 dilution) overnight at 4°C. On the following day, the cells were incubated with a fluorescently tagged secondary antibody (Jackson ImmunoResearch), stained with 0.1 μg·mL^−1^ 4′,6‐diamidino‐2‐phenylindole (DAPI) to identify the nucleus, and imaged using a fluorescence microscope (ECLIPSE TE2000‐U, Nikon).

### 2.8 RNA isolation, reverse transcription, and quantitative real‐time polymerase chain reaction

Total RNA was isolated from cells using TRIzol (Invitrogen) and reverse‐transcribed into cDNA using the PrimeScript RT Reagent Kit (Takara, Otsu, Japan). Quantitative real‐time polymerase chain reaction (qRT‐PCR) was performed using SYBR Green (Takara) with the FTC‐3000P system (Funglyn Biotech, Toronto, ON, Canada). miRNA and mRNA expression was normalized to U6 and β‐actin levels, respectively. The primers used are listed in Table S2. Each sample was assayed in triplicate and analyzed using the $2^{\left(-\Delta \Delta C_{t}\right)}$ method. Each experiment was independently performed three times.

### 2.9 ChIP and luciferase reporter assay

The promoter of miR‐373 (http://grch37.ensembl.org/index.html) was studied using the bioinformatics software jaspar (http://jaspar.genereg.net). For ChIP, cells were pretreated with 1% formaldehyde for crosslinking. After treatment with glycine to quench the reaction, the cells were resuspended sequentially in Mg‐NI, Mg‐NI‐XP40, Ca‐NI, and lysis buffer. Ultrasonication was performed to shear the DNA into fragments approximately 500 base pairs in length. The sheared chromatin was immunoprecipitated using anti‐CREB, anti‐p‐CREB, or anti‐IgG antibodies; subsequently, the ChIP products in each immunoprecipitation reaction were analyzed by PCR and agarose gel electrophoresis. The primers used are listed in Table S3. The sequence upstream of miR‐373 harboring the putative wild‐type or mutated CREB binding site (Table S4) was subcloned into the pGL3‐reporter vector (Promega, Madison, WI, USA) and used in the luciferase reporter assay. The cloned constructs and the blank pGL‐reporter vector were cotransfected with the CREB1 or CON plasmid. Luciferase activities were measured to determine promoter activation after 24 h. Experiment was performed three times independently.

### 2.10 Dual‐luciferase reporter assay

miR‐373 targets were predicted using the following online algorithms: RNA22 (https://cm.jefferson.edu/rna22/Interactive/), PicTar (http://pictar.mdc-berlin.de), and RegRNA (http://regrna.mbc.nctu.edu.tw/html/tutorial.html). The sequences encoding the wild‐type or mutated predicted binding sites of miR‐373 on APC or TIMP2 (Table S5) were synthesized and subcloned into pmirGLO (Promega). The wild‐type pmirGLO‐APC‐WT/TIMP2‐WT and the mutated pmirGLO‐APC‐MT/TIMP2‐MT constructs were cotransfected with the miR‐373 or miR‐Ctrl plasmids. Luciferase activities were expressed as the ratio of firefly to *Renilla* luciferase activity and normalized to the control using the Dual‐Luciferase Assay Kit (Promega).

### 2.11 *In vivo* experiments for xenograft tumor and tumor metastasis using nude mice

All *in vivo* assays were conducted under the guidelines of the Animal Care and Use Committee of Xi’an Jiaotong University. Nude mice were obtained from the Animal Center of Xi’an Jiaotong University. Lentivirus containing sponge‐miR‐373 was purchased from Hanbio Biotech (Shanghai, China) and used to sponge and stably inhibit intracellular miR‐373 (Ebert and Sharp, 2010). HCT116 cells were infected with the virus and screened with puromycin to generate the stable cell line HCT116‐sponge‐miR‐373. A control stable line, HCT116‐sponge‐miR‐Ctrl, was constructed in a similar manner.

For the tumor metastasis model, 2 × 10^6^ HCT116‐sponge‐miR‐373 or HCT116‐sponge‐miR‐Ctrl cells were injected into the tail veins of nude mice. After 40 days, bioluminescence in the surviving mice was imaged using the Xenogen Imaging System (Xenogen, Alameda, CA, USA). Subsequently, the mice were sacrificed, their lungs were removed, and metastases were confirmed by direct monitoring of the luciferase signals.

For the xenograft tumor model, 3 × 10^6^ cells were subcutaneously injected into the posterior flanks of nude mice. NE or saline was injected intraperitoneally every day. The length (*L*) and width (*W*) of the xenografts were measured every 3 days, and tumor volume (*V*) was calculated using the formula *V* = (*L* × *W*)^2^/2. On the last day, the xenografts were removed and weighed.

### 2.12 Bioinformatics analysis

Tyrosine hydroxylase (TH) has been used as a biomarker of adrenergic nerves (Faulkner *et al.*, 2019). We extracted data on TH, CREB1, hsa‐miR‐373‐3p, APC, and TIMP2 in tumor tissues from the RNAseq Illumina HiSeq dataset in the Cancer Genome Atlas (TCGA) of the University of California Santa Cruz Genomics Institute (https://xenabrowser.net/heatmap/; Cancer Genome Atlas Network, 2012). The dataset included 23 cases of TCGA colon cancer (COAD) and 14 cases of TCGA rectal cancer (READ) with simultaneous data on TH, CREB1, hsa‐miR‐373‐3p, APC, and TIMP2. The cases were ranked according to the level of expression of TH and divided into two groups; the half of the cases with lower TH expression formed the Low TH RNA level group, and the other half made up the High TH RNA level group. The data on CREB1, hsa‐miR‐373‐3p, APC, and TIMP2 in these two groups were analyzed using spss software (IBM SPSS, Chicago, IL, USA), and the unpaired *t*‐test was used to compare them.

### 2.13 Clinical tissue specimens

CRC tumor samples were obtained from the Third Affiliated Hospital of Xi’an Jiaotong University (Xi’an, China). None of the patients had received prior chemotherapy, radiotherapy, or systemic therapy, and none had additional malignant tumors. All samples were obtained with the informed consent of the patient prior to collection. The present study was approved by the Medical Ethics Committee of Xi’an Jiaotong University. The study methodologies conformed to the standards set by the Declaration of Helsinki.

### 2.14 Statistical analysis

Data are presented as mean ± standard deviation. Statistical analysis was performed using spss software; Student's *t*‐test was used to compare groups. *P* < 0.05 was considered statistically significant.

## 3 Results

### 3.1 NE promotes colon cancer cell proliferation, migration, and invasion via CREB1

**Fig. 1:**
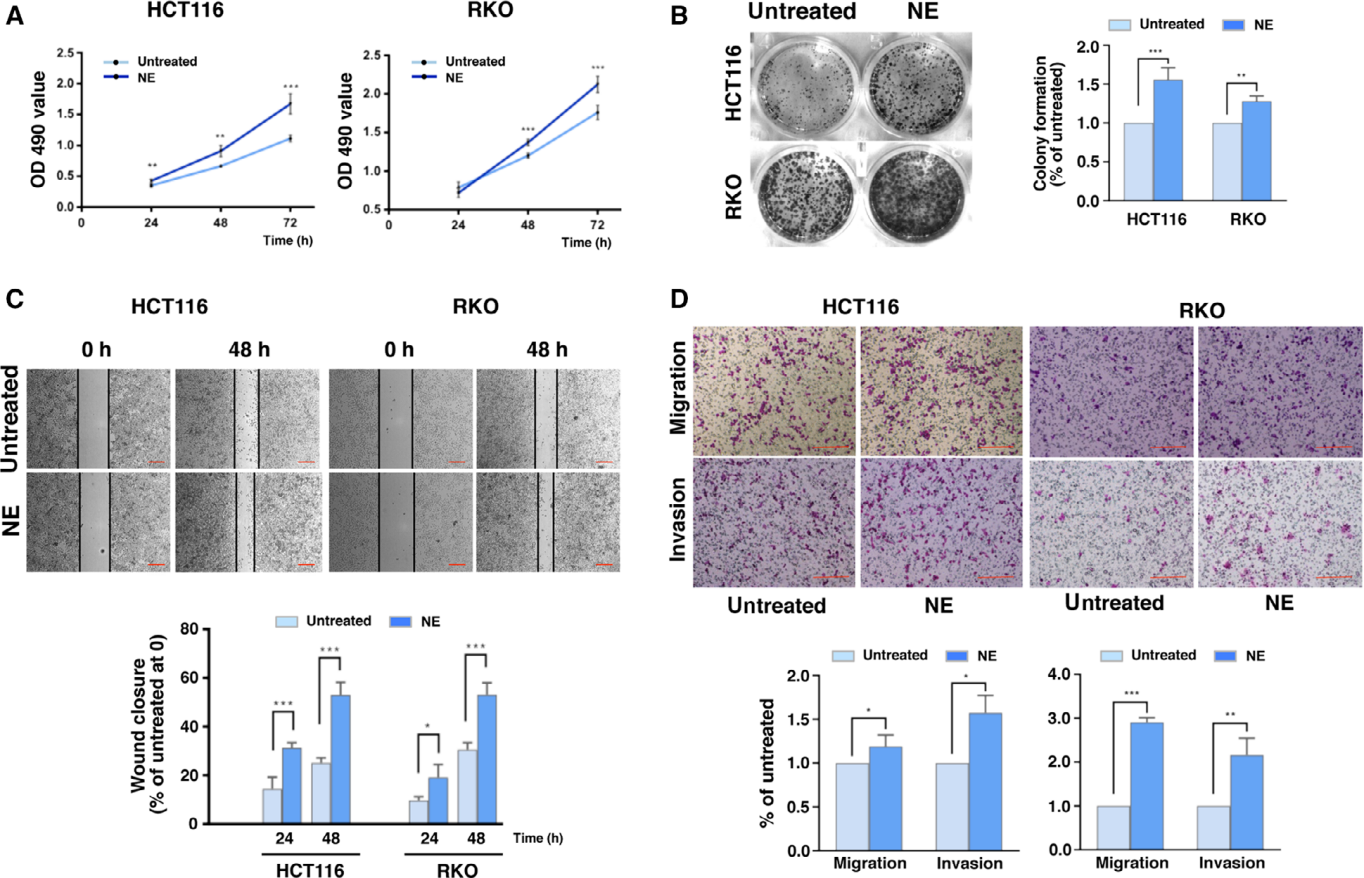
Norepinephrine (NE) promotes the proliferation, migration, and invasion of human colon cancer cells. (A) Human colon cancer cells (HCT116 and RKO) were treated with NE for 24, 48, and 72 h. Changes in cell viability were measured using MTT assays. (B) HCT116 and RKO cells were treated with NE for 1 week, after which the colony formation assay was performed. (C) Wound healing assays were performed in HCT116 and RKO cells treated with NE. Percentage of wound closure at different times was calculated as the ratio of the distance between the cells in the wound at each time point to that at 0 h. Scale bars represent 100 μm. (D) Transwell assay showing the migration and invasion of HCT116 and RKO cells treated with NE. Scale bars represent 100 μm. The experiments were independently performed three times and showed reproducible results. Error bars are represented as mean ± SD (*n* = 5). *P*‐values were calculated using Student's *t*‐test. * represents *P* < 0.05, ** represents *P* < 0.01, and *** represents *P* < 0.001.

We determined the effect(s) of NE on HCT116 and RKO cells. The results of the MTT assays showed that NE enhanced cell viability in both cell types compared to untreated cells (Fig. 1A, Fig. S1). The colony‐forming assay also demonstrated that NE stimulated colony formation (Fig. 1B). Moreover, the results of the wound healing assay showed that NE promoted wound closure (Fig. 1C). Since the wound healing assay results reflect both cell proliferation and cell migration, we used the transwell assay to confirm that NE increased the migratory and invasive nature of the HCT116 and RKO cells (Fig. 1D).

**Fig. 2:**
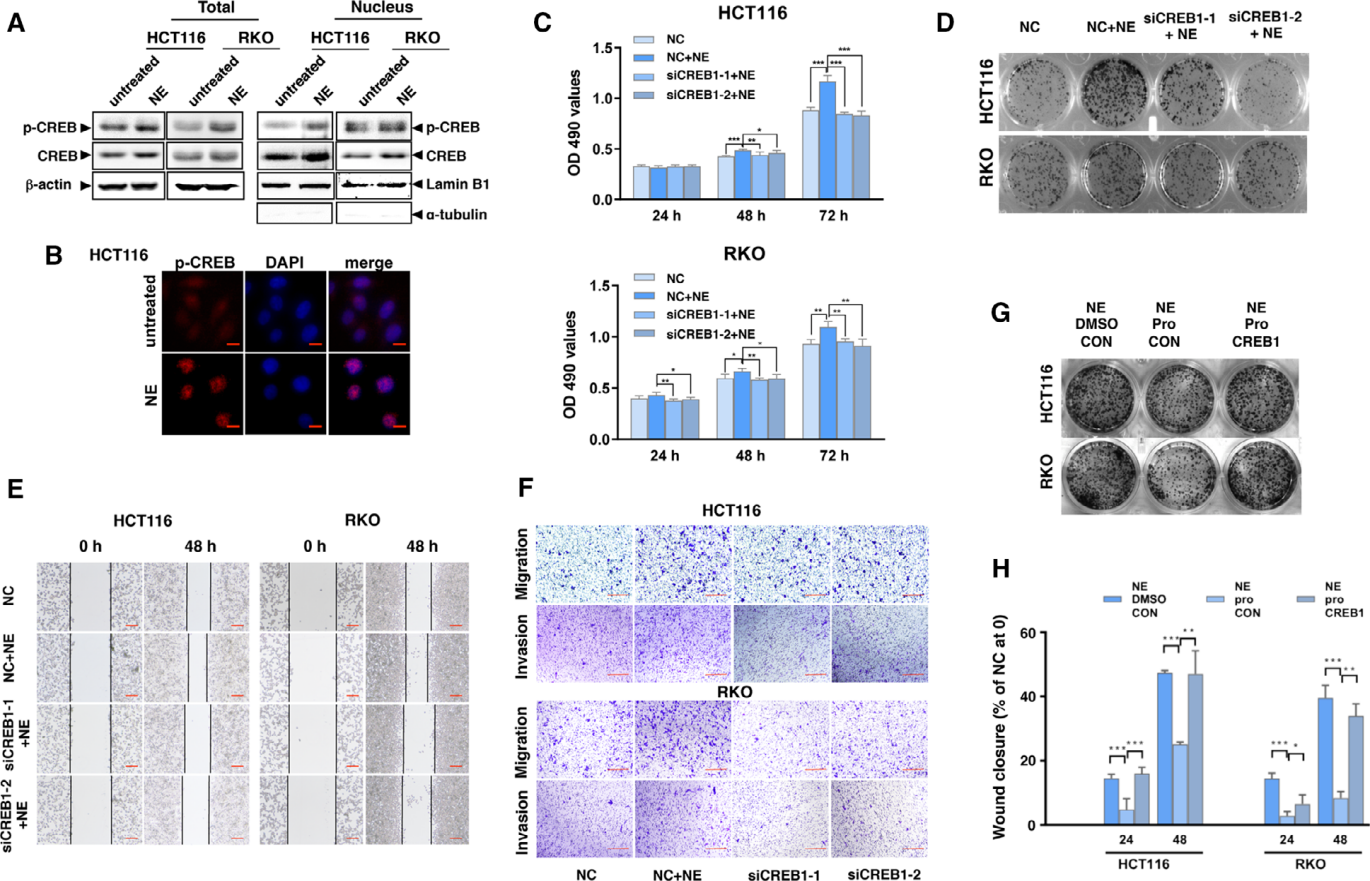
NE activates CREB and enhances proliferation, migration, and invasion of colon cancer cells. (A) HCT116 and RKO cells were treated with NE for 48 h. Protein levels of phospho‐CREB1 and CREB1 were detected in the nucleus and in whole‐cell lysates by western blotting using lamin B1 and β‐actin as loading controls. The blot with α‐tubulin in nuclear samples is presented to show the separation. (B) Representative images showing the expression of phospho‐CREB1 in HCT116 cells treated with NE (phospho‐CREB1 stained in red; nucleus stained with DAPI in blue). Scale bars represent 10 μm. (C) HCT116 and RKO cells were transfected with siCREB1‐1, siCREB1‐2, or the control (NC) and treated with NE for 24, 48, and 72 h. Changes in cell viability were detected by the MTT assay. (D) Results of colony formation assays. (E) Representative images of the wound healing assay. Scale bars represent 100 μm. (F) Representative images for the transwell assay showing the migration and invasion of the colon cancer cells. Scale bars represent 100 μm. In addition, HCT116 and RKO cells were transfected with CREB1 or the control (CON) followed by treatment with NE, propranolol (pro), or a combination of NE and pro. (G) Results of colony formation assay. (H) Results of wound healing assay. The experiments were independently performed three times with reproducible results. Error bars are represented as mean ± SD (*n* = 5). *P*‐values were calculated using Student's *t*‐test. * represents *P* < 0.05, ** represents *P* < 0.01, and *** represents *P* < 0.001.

cAMP response element‐binding protein is a mediator of stress‐induced maladaptation (Peng *et al.*, 2015); thus, we investigated its role in the cellular changes that occur in NE‐induced colon cancer. Western blotting and immunofluorescence assays showed that NE increased the levels of p‐CREB both in the nucleus and in whole‐cell extracts (Fig. 2A,B, Fig. S2A). We used siCREB1‐1 and siCREB1‐2 individually to inhibit CREB1 expression in HCT116 and RKO cells (Fig. S3A). The MTT and colony‐forming assays showed that this reduced the previously observed NE‐induced proliferation of cells (Fig. 2C,D, Fig. S2B). Moreover, siCREB1 activity in the NE‐treated cells delayed wound closure and inhibited migration and invasion (Fig. 2E,F, Fig. S2C,D). We utilized propranolol, an inhibitor of β‐AR (Benish *et al.*, 2008), to block NE and found that this decreased NE‐induced colony formation and wound closure and that this effect was rescued by overexpression of CREB1 (Fig. 2G,H, Fig. S3B). These results suggest that NE promotes cell proliferation, migration, and invasion by activating CREB1 in human colon cancer cells.

### 3.2 NE increases miR‐373 expression via CREB1

**Fig. 3:**
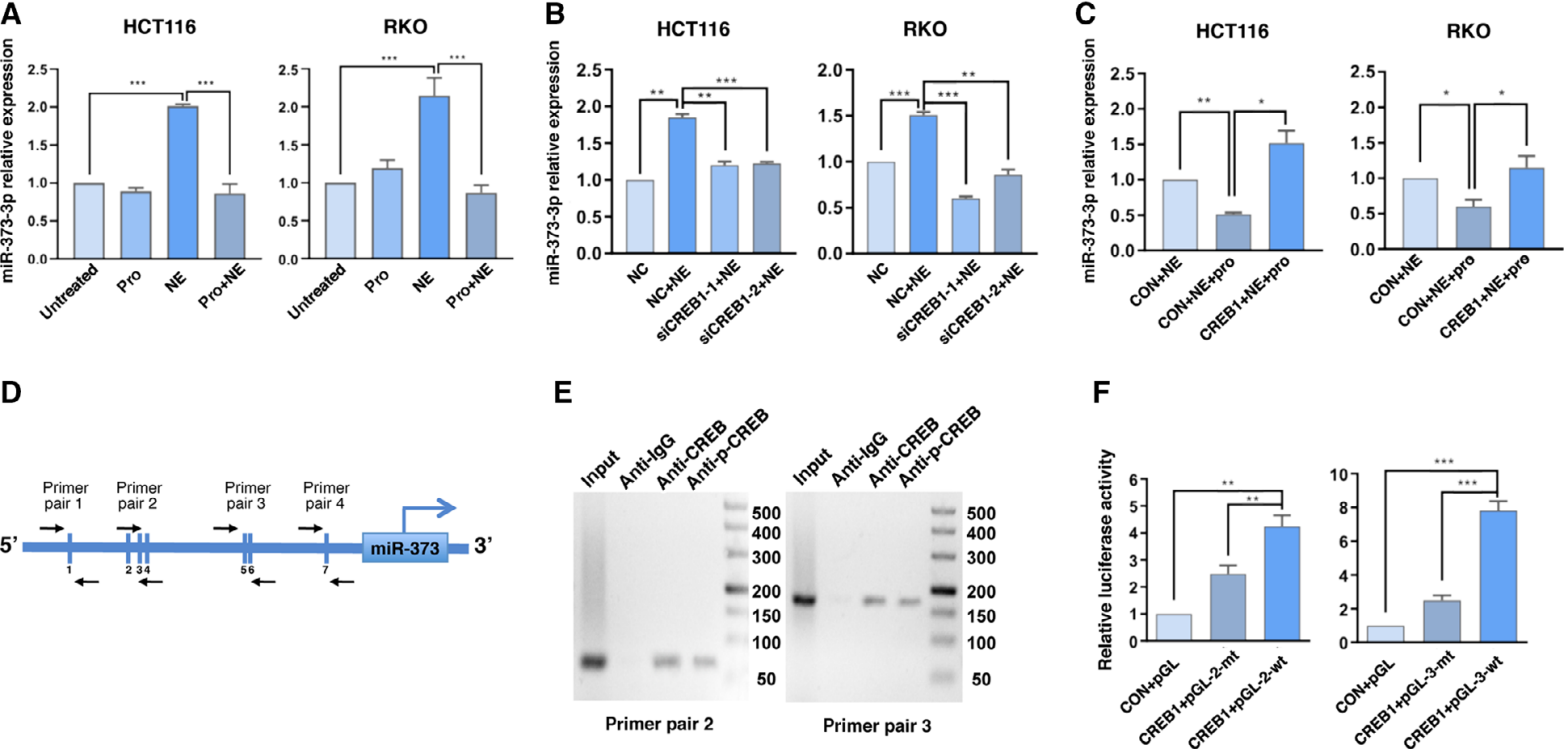
CREB1 transcriptionally activates miR‐373 following NE treatment. (A) HCT116 and RKO cells were treated with NE, pro, a combination of NE and pro or left untreated for 48 h. Changes in miR‐373‐3p expression were detected using qRT‐PCR. (B) Changes in the expression of miR‐373‐3p in HCT116 and RKO cells transfected with siCREB1‐1, siCREB1‐2, or the control (NC) followed by treatment with NE for 48 h. (C) HCT116 and RKO cells were transfected with CON or CREB1 followed by NE or a combination of NE and pro for 48 h. Changes in miR‐373‐3p were detected using qRT‐PCR. (D) Analysis of the promoter of miR‐373 using JASPAR. Four pairs of primers were designed and synthesized with putative CREB binding sites. (E) ChIP assays were performed to detect the occupancy of CREB1 on the miR‐373 promoter. (F) Luciferase reporter assays were performed to determine the binding of CREB1 to the miR‐373 promoter. Experiments were independently performed three times with reproducible results. Error bars are represented as mean ± SD (*n* = 4). *P*‐values were calculated using Student's *t*‐test. * represents *P* < 0.05, ** represents *P* < 0.01, and *** represents *P* < 0.001.

Previous studies have shown that the expression of miR‐373 is regulated by CREB in zinc transporter 4‐induced progression of pancreatic cancer (Awasthi *et al.*, 2018). Since microRNAs elicit specific cellular effects (Esteller, 2011), we wanted to understand the role of miR‐373 in our experimental setup. qRT‐PCR showed that, compared to the untreated cells, NE increased miR‐373 levels, whereas propranolol inhibited miR‐373 expression (Fig. 3A). Moreover, siCREB1‐1 and siCREB1‐2 inhibited the NE‐induced increase in miR‐373 levels (Fig. 3B). Propranolol inhibited the NE‐induced expression of miR‐373, and overexpression of CREB1 reversed the effects of propranolol (Fig. 3C). Only overexpression of CREB1 enhanced miR‐373 expression; siCREB1‐1 and siCREB1‐2 inhibited miR‐373 expression (Fig. S3C, Fig. S3D). These data suggest that NE transcriptionally upregulates miR‐373 via CREB1.

### 3.3 CREB1 transcriptionally activates miR‐373 expression

Next, we analyzed the upstream sequences of miR‐373 (http://grch37.ensembl.org/index.html) to discover CREB binding sites using JASPAR (http://jaspar.genereg.net). The seven putative CREB binding site cassettes are schematically represented by vertical sticks in Fig. 3D and Table S3. Five of these sites (binding sites 2, 3, and 4 and binding sites 5 and 6) were clustered in two regions. We synthesized two pairs of primers (primer pair 2 and primer pair 3) for the clustered regions and two primer pairs (primer pair 1 and primer pair 4) for the other two binding sites (binding site 1 and binding site 7). Subsequently, we performed ChIP to confirm the binding of CREB to these two regions (Fig. 3E). To determine whether these two regions are directly involved in miR‐373 regulation, we generated two pGL‐wt constructs containing the putative CREB binding sites and two pGL‐mt plasmids containing mutated versions of the CREB binding sites (Table S4) and used these in the luciferase reporter assay. Compared with the control constructs, cotransfection of the CREB1 and pGL‐wt plasmids resulted in high luciferase activity, whereas cotransfection of the CREB1 and pGL‐mt plasmids resulted in near‐basal luciferase activity (Fig. 3F). Therefore, CREB1 binds to the promoter of miR‐373 and activates the transcription of miR‐373 in colon cancer cells.

### 3.4 NE enhances colon cancer cell proliferation, migration, and invasion via miR‐373

miR‐373 inhibits migration and invasion of glioblastoma (Gao *et al.*, 2018; Jing *et al.*, 2017), but it functions as an oncogene in other cancers (Liu *et al.*, 2015; Voorhoeve *et al.*, 2006; Weng *et al.*, 2017; Zhang *et al.*, 2013). Thus, we wished to determine the role of miR‐373 in colon cancer. We constructed a plasmid that overexpresses miR‐373 and confirmed its efficiency (Fig. S4A). Overexpression of miR‐373 increased cell proliferation (Fig. S5A,B), whereas inhibition of endogenous miR‐373 using an miR‐373 inhibitor (Fig. S4B) decreased cell proliferation (Fig. S4A,B). The wound healing assay showed that overexpression of miR‐373 enhanced wound closure, while the miR‐373 inhibitor hindered the process of wound closure (Fig. S5C). These results were confirmed *in vivo* using mice. We generated the HCT116‐sponge‐miR‐373 cell line wherein the function of miR‐373 was stably inhibited (Ebert and Sharp, 2010); HCT116‐sponge‐miR‐Ctrl cells were used as a control. We injected these cells into mice via the tail vein and monitored metastasis by imaging 40 days later. Three of four mice injected with the HCT116‐sponge‐miR‐Ctrl cells developed metastatic foci in the lungs; however, only one of four mice injected with the HCT116‐sponge‐miR‐373 cells developed metastatic foci (Fig. S4D). These results demonstrate that oncogenic miR‐373 induces the proliferation and metastasis of human colon cancer cells.

**Fig. 4:**
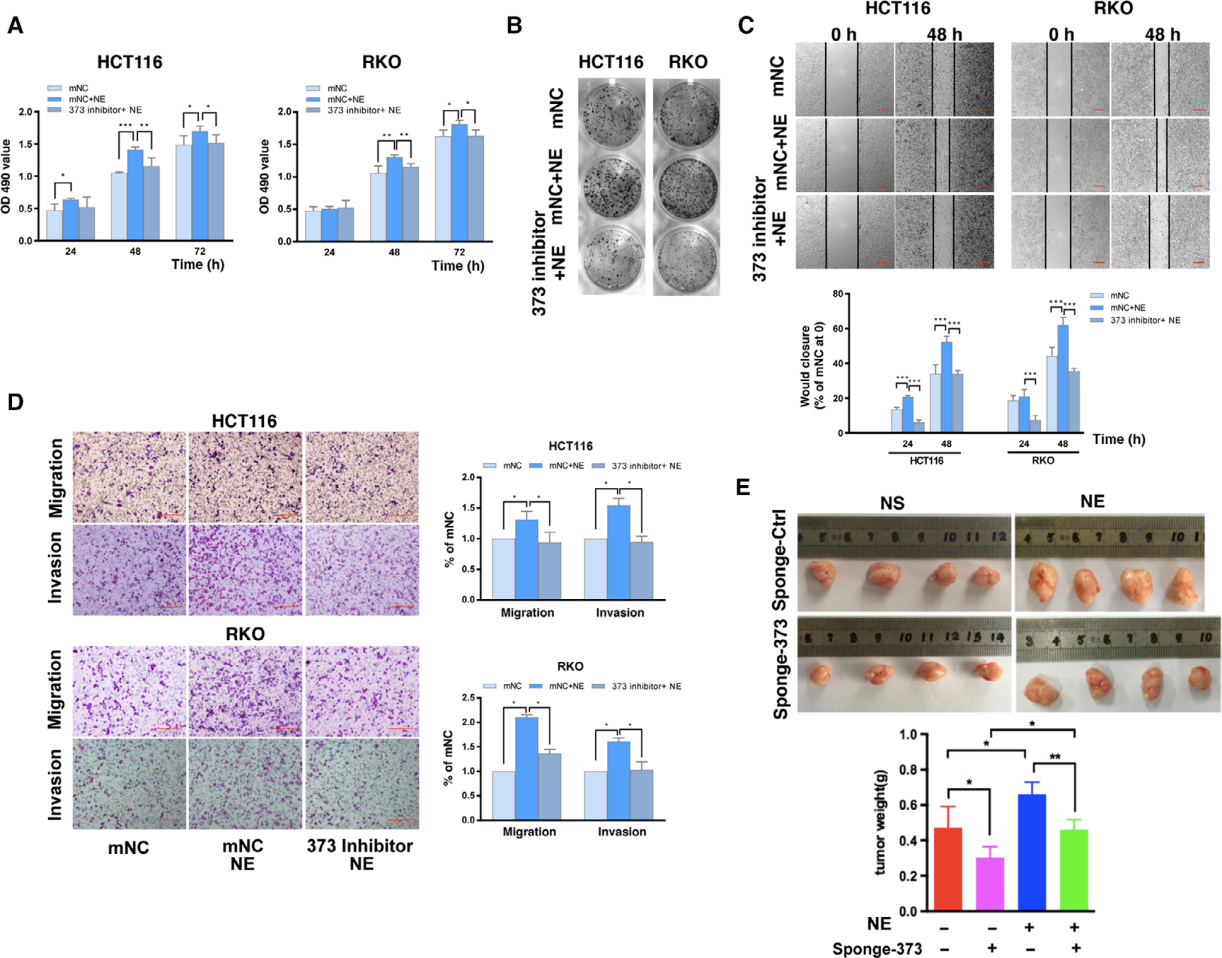
NE stimulates the progression of colon cancer by miR‐373 *in vitro* and *in vivo*. (A) HCT116 and RKO cells were transfected with miR‐373 inhibitor (373 inhibitor) or the control (mNC) and treated with NE for 24, 48, and 72 h. Changes in cell viability were detected by the MTT assay. (B) Colony formation assay. (C) Representative images and quantification of the wound healing assay. Scale bars represent 100 μm. (D) Representative images and calculated percentages of migrating cells in the transwell assay for migration and invasion. Scale bars represent 100 μm. (E) HCT116/sponge‐miR‐Ctrl or HCT116/sponge‐miR‐373 cells (3 × 10^6^) were subcutaneously injected into the posterior flanks of nude mice; NE or saline was then intraperitoneally injected every day. Three weeks later, the orthotopic xenografts were removed. Representative images from each group are shown. The orthotopic tumors were weighed. The experiments were independently performed three times with reproducible results. Error bars are represented as mean ± SD (*n* = 5). *P*‐values were calculated using Student's *t*‐test. * represents *P* < 0.05, ** represents *P* < 0.01, and *** represents *P* < 0.001.

Next, we explored whether miR‐373 is involved in the phenotypic differences induced by NE. NE‐induced cell proliferation was suppressed by transfection with the miR‐373 inhibitor (Fig. 4A,B). The results of the wound healing and transwell assays showed that the miR‐373 inhibitor reduced NE‐induced cell migration and invasion (Fig. 4C,D). Thus, NE promoted cell proliferation, migration, and invasion via miR‐373 in colon cancer cells. These results were then tested in the xenograft tumor model. Mice were divided into 4 groups: normal saline (NS)+sponge‐miR‐Ctrl, NE + sponge‐miR‐Ctrl, NS + sponge‐miR‐373, and NE + sponge‐miR‐373. All the mice were healthy and of equal body weight (Fig. S6A). NE treatment increased the tumor size and weight, whereas sponge‐miR‐373 significantly restricted the progression of the NE‐induced xenografts (Fig. 4E, Fig. S6B). These results indicate that NE promotes proliferation, migration, and invasiveness in colon cancer via miR‐373.

### 3.5 TIMP2 and APC are targets of miR‐373 in human colon cancer cells

**Fig. 5:**
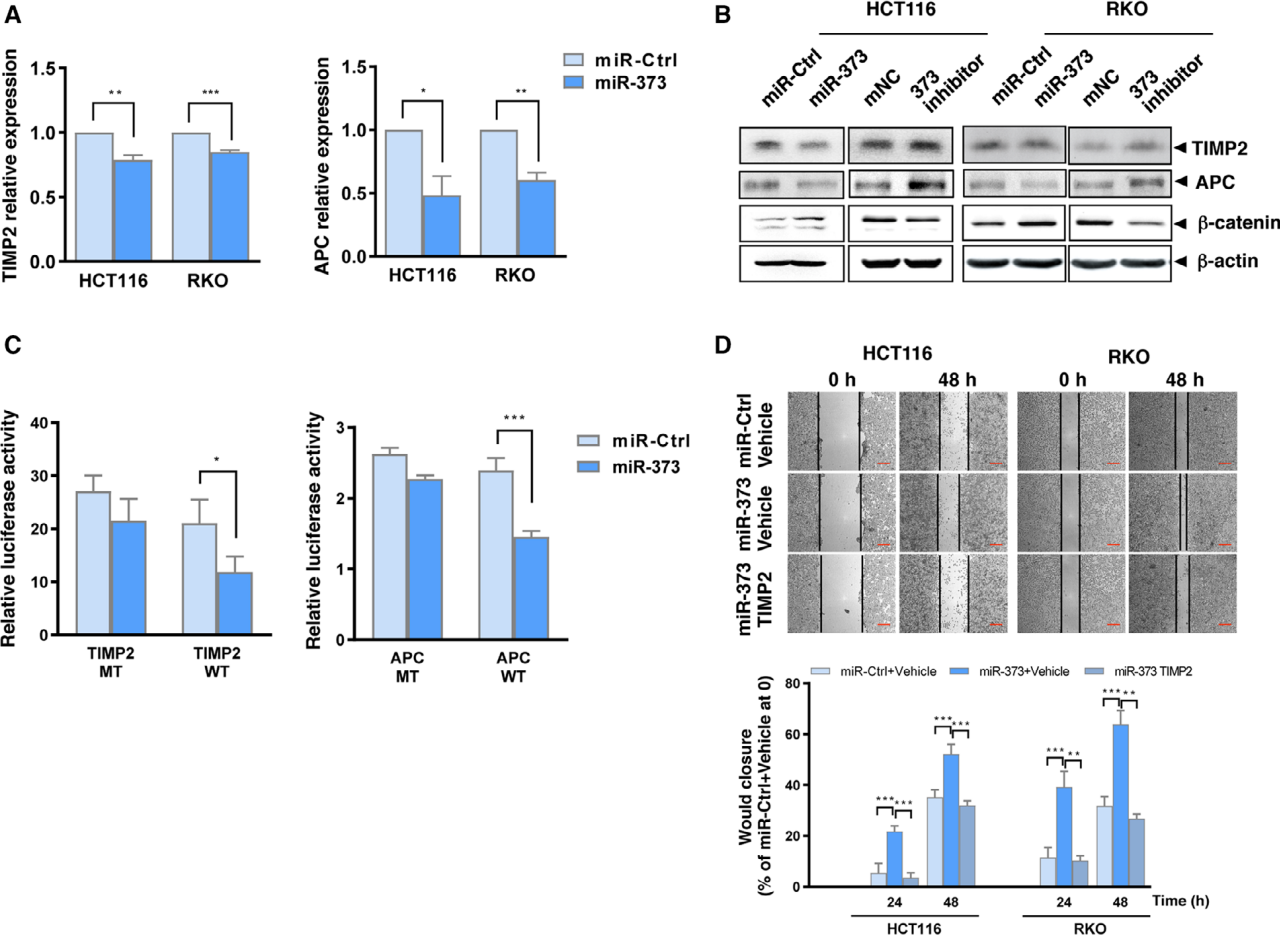
miR‐373 targets TIMP2 and APC. (A) mRNA levels of TIMP2 and APC as analyzed by qRT‐PCR in HCT116 and RKO cells transfected with miR‐373 or miR‐Ctrl. (B) Protein levels of TIMP2, APC, and β‐catenin in HCT116 and RKO cells transfected with miR‐373, miR‐Ctrl, miR‐373 inhibitor, or mNC were detected by western blotting. β‐actin levels were used as the loading control. (C) The luciferase reporter assay was performed in HCT116 cells containing miR‐373 or miR‐Ctrl cotransfected with pmirGLO‐wild‐type (TIMP2‐WT or APC‐WT) or pmirGLO‐mutant constructs (TIMP2‐MT or APC‐MT). (D) HCT116 and RKO cells were transfected with miR‐Ctrl, miR‐373, or miR‐373 and the TIMP2 plasmid. Subsequently, wound healing assays were performed and quantified. Representative images and percentage of wound closure are shown. Scale bars represent 100 μm. The experiments were independently repeated three times with reproducible results. Error bars are represented as mean ± SD (*n* = 5). *P*‐values were calculated using Student's *t*‐test. * represents *P* < 0.05, ** represents *P* < 0.01, and *** represents *P* < 0.001.

MicroRNAs are noncoding RNAs that degrade target mRNAs or inhibit translation through base pairing with target genes (Esteller, 2011). We used common bioinformatics tools (RNA22, picTAR, and RegRNA) to predict the targets of miR‐373. TIMP2 and APC, among many others, were found to be novel targets of miR‐373 among many others (Fig. S7A). We validated the changes in TIMP2 and APC expression by modulating the levels of miR‐373. The mRNA levels of TIMP2 and APC decreased upon overexpression of miR‐373 (Fig. 5A). The protein levels of TIMP2 and APC also decreased upon miR‐373 overexpression and increased using the miR‐373 inhibitor (Fig. 5B). APC is a critical component of the β‐catenin degradation complex (Kohler *et al.*, 2009); consistent with this, miR‐373 overexpression upregulated β‐catenin expression, whereas exposure of the cells to the miR‐373 inhibitor downregulated β‐catenin expression (Fig. 5B). We used an immunofluorescence assay to discover that the fluorescence intensity imparted by APC in miR‐373‐overexpressing cells was significantly lower than that seen in the control cells (Fig. S7B). These results suggest that miR‐373 downregulates TIMP2 and APC. We then generated wild‐type and mutated versions of TIMP2 and APC (TIMP2‐WT, APC‐WT, TIMP2‐MT, and APC‐MT) using the pmirGLO backbone (Table S5) and used them in the dual‐luciferase reporter assay. miR‐373 overexpression decreased the luminescence observed when the TIMP2‐WT or APC‐WT constructs were used compared to that observed in miR‐Ctrl cells, whereas there were no differences in the amount of luminescence generated by the TIMP2‐MT or APC‐MT constructs in cells cotransfected with miR‐373 and miR‐Ctrl (Fig. 5C). These results indicate that TIMP2 and APC are direct targets of miR‐373.

### 3.6 miR‐373–TIMP2 mediates NE‐induced colon cancer cell proliferation, migration, and invasion

**Fig. 6:**
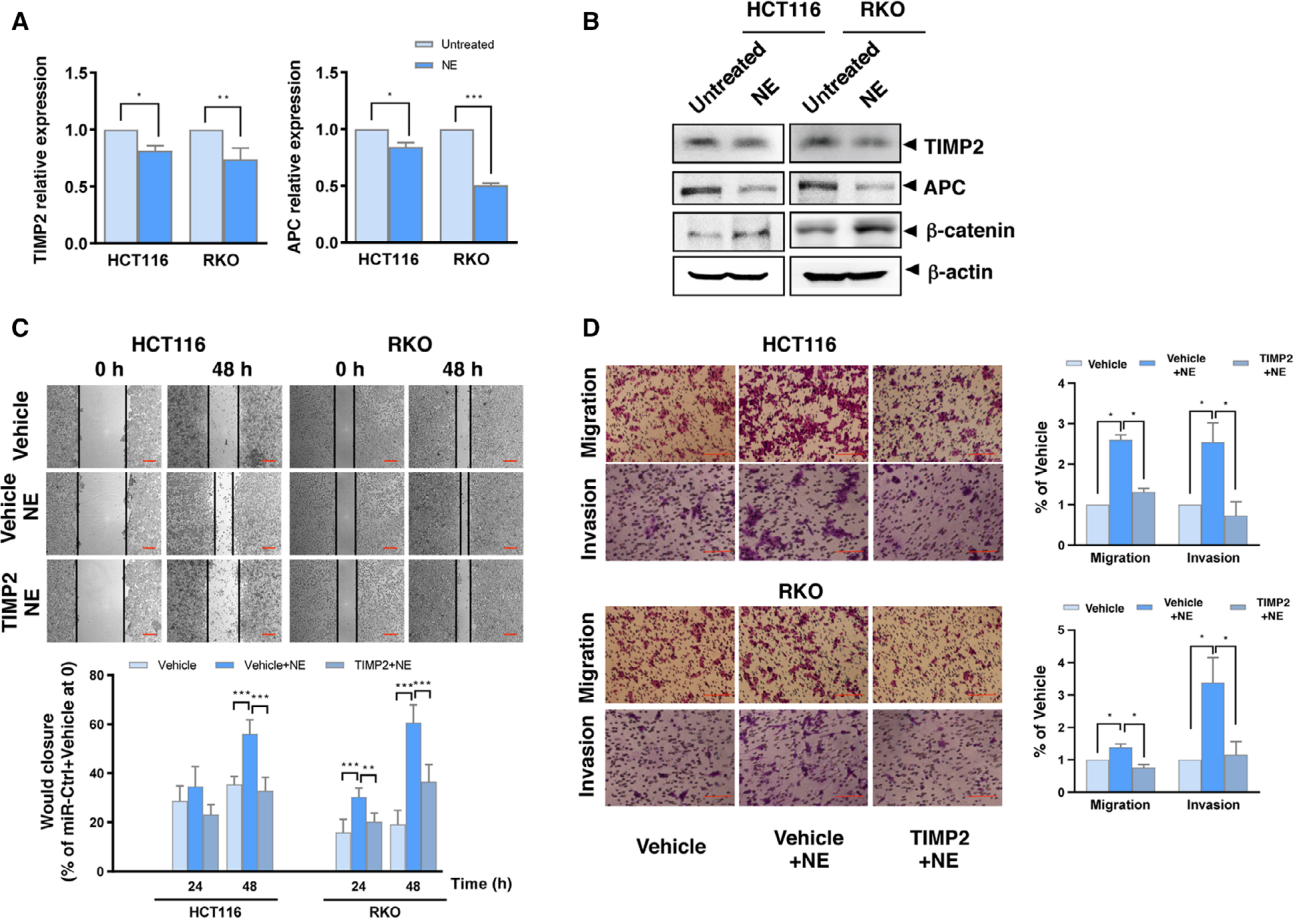
miR‐373‐target genes mediate NE‐induced effects. (A) The mRNA levels of TIMP2 and APC were analyzed using qRT‐PCR in HCT116 and RKO cells treated with NE. (B) The protein levels of TIMP2, APC, and β‐catenin in HCT116 and RKO cells treated with NE were detected by western blotting. β‐actin was used as the loading control. (C) HCT116 and RKO cells were transfected with TIMP2 or vehicle plasmids followed by NE treatment. Representative images and quantification of the wound healing assay are shown. Scale bars represent 100 μm. (D) Representative images and percentage migration in the transwell assay for migration and invasion. Scale bars represent 100 μm. The experiments were independently repeated three times with reproducible results. Error bars are represented as mean ± SD (*n* = 5). *P*‐values were calculated using Student's *t*‐test. * represents *P* < 0.05, ** represents *P* < 0.01, and *** represents *P* < 0.001.

A role for APC in the development of colon cancer has been reported (Lesko *et al.*, 2014); thus, we focused on TIMP2. We generated a TIMP2‐overexpressing plasmid and observed that overexpression of TIMP2 significantly inhibited miR‐373‐induced wound closure (Fig. 5D, Fig. S7C). This indicates that miR‐373‐induced cell proliferation and migration is mediated at least in part by TIMP2. We then verified the roles of the miR‐373 targets in mediating the NE‐induced effects. NE decreased the mRNA and protein levels of TIMP2 and APC but increased the protein levels of β‐catenin (Fig. 6A,B). The wound healing assay showed that TIMP2 overexpression reduced NE‐induced wound closure (Fig. 6C). Similarly, TIMP2 overexpression also inhibited NE‐induced cell migration and invasion (Fig. 6D). These results suggest that miR‐373 targets mediate the NE‐induced phenotype in HCT116 and RKO cells.

### 3.7 NE‐CREB1‐miR‐373 signaling axis in CRC

**Fig. 7:**
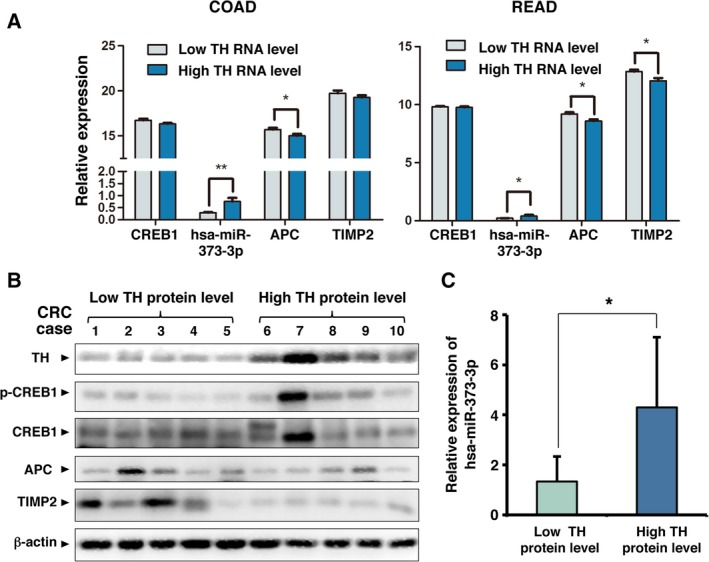
Analysis of TH, CREB1, hsa‐miR‐373‐3p, APC, and TIMP2 expression in human CRC using TCGA database and clinical CRC samples. (A) Data on TH, CREB1, hsa‐miR‐373‐3p, APC, and TIMP2 expression in CRC tumor tissues were extracted from the RNAseq Illumina HiSeq dataset in TCGA database. There were 23 cases of TCGA colon cancer (COAD) and 14 cases of TCGA rectal cancer (READ) with simultaneous data on TH, CREB1, hsa‐miR‐373‐3p, APC, and TIMP2. The cases were ranked according to the expression level of TH and divided into two groups; the half with lower TH expression was designated the Low TH RNA level group, and the other half was designated the High TH RNA level group. The expression of CREB1, hsa‐miR‐373‐3p, APC, and TIMP2 in these two groups was compared, and *P*‐values were calculated using unpaired *t*‐test. (B) Protein lysates were harvested from the tumors of each clinical CRC case. The levels of TH, phospho‐CREB1, CREB1, APC, and TIMP2 were detected by western blotting. β‐actin was used as the loading control. (C) RNA was extracted from the tumors of clinical CRC patients. The levels of hsa‐miR‐373‐3p were measured by qRT‐PCR and compared between the group with Low TH protein level and the group with High TH protein level. Error bars are represented as mean ± SD (*n* = 12). *P*‐values were calculated using unpaired *t*‐test. * represents *P* < 0.05 and ** represents *P* < 0.01.

We extracted gene expression RNAseq data from the TCGA database (Fig. 7A). Since tyrosine hydroxylase (TH) has been used as a biomarker of adrenergic nerves (Faulkner *et al.*, 2019), we divided the data into two groups, a group with Low TH RNA levels (representing CRC tumors with low adrenergic innervation) and a group with High TH RNA level, indicating highly adrenergically innervated CRC tumors. Although the two groups did not differ in their CREB1 RNA levels, the level of miR‐373 was higher in the High TH group of colon cancer (COAD) and rectal cancer (READ) than in the Low TH group. APC RNA was lower in the High TH RNA group than in the Low TH RNA group. TIMP2 RNA was lower in the High TH RNA group of READ than in the Low TH group. In COAD, there was also a tendency toward a decrease in TIMP2 RNA in the High TH RNA group compared with that in the Low TH RNA group. In addition, we harvested proteins from clinical CRC tumor tissues (Fig. 7B). The western blotting results indicated that the protein levels of p‐CREB were higher in CRC tissue samples with relatively high levels of TH protein than in CRC tissue samples with relatively low levels of TH protein. In addition, the protein levels of APC and TIMP2 were lower in CRC tissue samples with relatively high levels of TH protein than in CRC samples with relatively low expression of TH protein. hsa‐miR‐373‐3p was significantly higher in CRC tissue samples with relatively high levels of TH protein than in CRC samples with relatively low levels of TH protein (Fig. 7C). These results from public databases and clinical CRC patients are consistent with our *in vitro* and *in vivo* findings.

**Fig. 8:**
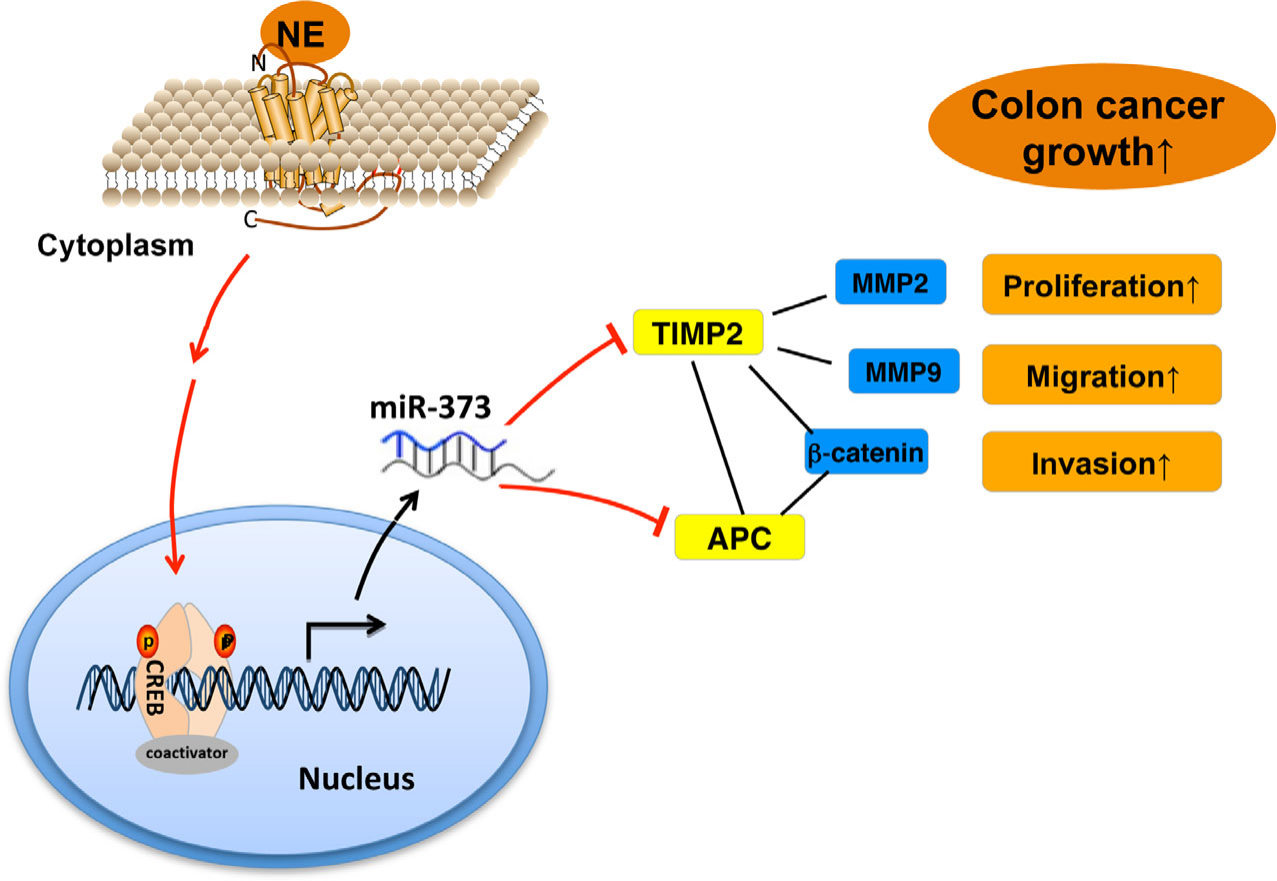
Schematic for NE–CREB1–miR‐373‐mediated progression of human colon cancer. NE increases the transcription of miR‐373 via CREB1, which represses the expression of the miR‐373 targets TIMP2 and APC, thereby resulting in increased cell proliferation, migration, invasion, and progression of human colon cancer.

Based on these results, we propose a novel NE–CREB1–miR‐373 signaling axis in human colon cancer cells (Fig. 8). We have demonstrated that NE promotes the progression of colon cancer via CREB1 activity‐dependent transcription, enhancing the expression of oncogenic miR‐373 and reducing the expression of the miR‐373 targets APC and TIMP2. This novel NE‐CREB1‐miR‐373 signaling axis may potentially serve as a new therapeutic target for CRC, which occurs commonly and is highly innervated by autonomic fibers, ultimately resulting in reduced patient survival (Liebl *et al.*, 2013).

## 4 Discussion

Organisms are continuously exposed to a variety of stresses that cause maladaptation and can even lead to cancer. In addition to changes in humoral immunity, recent studies have shown that the peripheral nerves in TNCs are associated with stress‐induced physiological changes in cancer cells (Arese *et al.*, 2018; Faulkner *et al.*, 2019; Jobling *et al.*, 2015; Magnon, 2015; Mauffrey *et al.*, 2019; Tang *et al.*, 2013). Neurotransmitters are age‐old secreted modulators that are evolutionarily conserved. They are widespread in non‐neural cancer tissues, including CRC (Huh *et al.*, 2010; Liebl *et al.*, 2013). An active adrenergic system is responsible for stress‐induced cancer progression; epidemiological evidence has shown that β‐blockers improve the long‐term survival of cancer patients (Coelho *et al.*, 2015). NE is secreted primarily by adrenergic fibers. Our previous report showed that chronic exposure to scream sound stress caused an increase in the levels of NE in the serum and progression of colon cancer in mice (Hou *et al.*, 2013). In this study, we have demonstrated a novel pathway (NE–CREB1–miR‐373 signaling) through which NE directly stimulates the progression of colon cancer.

Norepinephrine regulates cell proliferation, survival, and tumor progression via the β‐AR–cAMP–PKA axis in various cancer cells (Chida *et al.*, 2008). NE upregulates p‐CREB during neuronal plasticity (Yaniv *et al.*, 2008) and suppresses cytotoxicity by activating cAMP/PKA and CREB in human microglia‐like THP‐1 cells (Yang *et al.*, 2012). However, very little is known about the NE–CREB correlation in cancer. In this study, we used human colon cancer cells to show that NE activates CREB1. Inhibition of CREB1 in NE‐treated cells decreased cell proliferation and invasion, and the presence of CREB1 was crucial in the NE‐induced progression of colon cancer.

Previous studies have shown that microRNAs are transcriptionally regulated by CREB in neural tissues (Tan *et al.*, 2012; Xia *et al.*, 2017). In this study, we demonstrated that CREB1 promotes miR‐373 expression by binding to its promoter in human colon cancer cells. miR‐373 plays a myriad of roles in tumorigenesis and in the progression of tumors. miR‐373 targets the epidermal growth factor receptor and is a tumor suppressor in glioblastoma cells. However, miR‐373 is a potential oncogene in tongue squamous cell, testicular germ cell, esophageal squamous cell, and prostate cancers (Jing *et al.*, 2017; Voorhoeve *et al.*, 2006; Wei *et al.*, 2015; Weng *et al.*, 2017). We found that miR‐373 elicits oncogenic effects in human colon cancer cells and that it is correlated with the NE‐induced progression of colon cancer.

In this study, we showed that miR‐373 promotes the proliferation and metastasis of colon cancer both *in vitro* and *in vivo*. Metastases are the primary cause of cancer mortality, and matrix metalloproteinases and TIMPs play significant roles in tumor invasion and metastasis (Curran *et al.*, 2004). *TIMP2* is a tumor suppressor gene (Stetler‐Stevenson, 2008); its overexpression decreases metastasis in various cancers (Kai *et al.*, 2016; Stetler‐Stevenson, 2008). Interestingly, we found TIMP2 to be a target of miR‐373 and showed that both NE and miR‐373 downregulate TIMP2 expression; TIMP2 overexpression reversed the effects of miR‐373 and NE. To the best of our knowledge, this is the first report demonstrating that TIMP2 is the downstream effector of the NE–CREB1–miR‐373 axis that mediates metastasis in response to NE in colon cancer cells.

We also showed that APC is a target of miR‐373. *APC* is a tumor suppressor gene that modulates cellular processes such as cell proliferation and migration (Lesko *et al.*, 2014). Mutations in *APC* aberrantly activate the β‐catenin signaling that is involved in CRC tumorigenesis (Kohler *et al.*, 2009; Roose *et al.*, 1999). In our study, we used HCT116 and RKO cells that express wild‐type APC (Fukuyama *et al.*, 2008) to show that the mRNA and protein levels of APC were decreased by exposure to NE or by miR‐373 overexpression. These findings are similar to the previously reported effects of NE and miR‐373 (Kohler *et al.*, 2009; Lesko *et al.*, 2014; Roose *et al.*, 1999). In further support of our findings, previous studies have reported that TIMP2 inhibits Wnt/β‐catenin signaling in melanoma (Xia and Wu, 2015).

In addition to directly promoting the growth of cancer cells, NE has been reported to be associated with increased expression of vascular endothelial growth factor and with the development of abundant tumor vascularization that facilitates tumor progression (Faulkner *et al.*, 2019; Magnon, 2015; Thaker *et al.*, 2006). We also detected it in our experiments (unpublished results). This needs further investigation.

## 5 Conclusions

Our study reveals that NE promotes colon cancer cell proliferation and metastasis by activating the NE–CREB1–miR‐373 axis and demonstrates that the targets of miR‐373, TIMP2 and APC, mediate these NE‐induced effects. This novel signaling axis may provide mechanistic insights into the neural regulation of CRC and help in the design of future clinical studies on stress biology in CRC.

## Conflict of interest

The authors declare no conflict of interest.

## Author contributions

JH, NH, and CH designed the experiments. JH, QJ, RM, HZ, KT, LN, JM, YY, and FW performed the experiments. BD provided the tumor tissues of clinical CRC patients. XW, DT, and YC analyzed the data. JH and MW provided the reagents and analytical tools. JH and NH wrote the paper.

## Supporting information


**Fig. S1.** Viability changes of HCT116 and RKO induced by NE.
**Fig. S2.** Quantification results of Fig. 2.
**Fig. S3.** Changes of miR‐373‐3p induced by CREB1 overexpression and siCREB1.
**Fig. S4.** Changes of miR‐373‐3p induced by miR‐373 overexpression and miR‐373 inhibitor.
**Fig. S5.** miR‐373 promotes proliferation and metastasis in colon cancer.
**Fig. S6.** Changes of body weight and xenograft volume in experiments of Figure 4E.
**Fig. S7.** Putative miR‐373‐binding sites exists in the 3’‐UTR of TIMP2 and APC, representative images of APC in HCT116 cells transfected with miR‐373 or miR‐Ctrl, and changes of TIMP2 by TIMP2 overexpression.

**Table S1.** Sequences of interference oligonucleotides.
**Table S2.** Primers for qRT‐PCR.
**Table S3.** Primer pairs used in ChIP assay.
**Table S4.** Insert sequence of pGL3‐promoter plasmids.
**Table S5.** Insert sequences of pmirGLO plasmids.

## References

[mol212657-bib-0001] Amit M , Na'ara S and Gil Z (2016) Mechanisms of cancer dissemination along nerves. Nat Rev Cancer 16, 399–408. 27150016 10.1038/nrc.2016.38

[mol212657-bib-0002] Arese M , Bussolino F , Pergolizzi M , Bizzozero L and Pascal D (2018) Tumor progression: the neuronal input. Ann Transl Med 6, 89. 29666812 10.21037/atm.2018.01.01 PMC5890045

[mol212657-bib-0003] Awasthi R , Rathbone MJ , Hansbro PM , Bebawy M and Dua K (2018) Therapeutic prospects of microRNAs in cancer treatment through nanotechnology. Drug Deliv Transl Res 8, 97–110. 29185148 10.1007/s13346-017-0440-1

[mol212657-bib-0004] Benish M , Bartal I , Goldfarb Y , Levi B , Avraham R , Raz A and Ben‐Eliyahu S (2008) Perioperative use of beta‐blockers and COX‐2 inhibitors may improve immune competence and reduce the risk of tumor metastasis. Ann Surg Oncol 15, 2042–2052. 18398660 10.1245/s10434-008-9890-5 PMC3872002

[mol212657-bib-0005] Cancer Genome Atlas Network (2012) Comprehensive molecular characterization of human colon and rectal cancer. Nature 487, 330–337. 22810696 10.1038/nature11252 PMC3401966

[mol212657-bib-0006] Chida Y , Hamer M , Wardle J and Steptoe A (2008) Do stress‐related psychosocial factors contribute to cancer incidence and survival? Nat Clin Pract Oncol 5, 466–475. 18493231 10.1038/ncponc1134

[mol212657-bib-0007] Coelho M , Moz M , Correia G , Teixeira A , Medeiros R and Ribeiro L (2015) Antiproliferative effects of β‐blockers on human colorectal cancer cells. Oncol Rep 33, 2513–2520. 25812650 10.3892/or.2015.3874

[mol212657-bib-0008] Coelho M , Soares‐Silva C , Brandão D , Marino F , Cosentino M and Ribeiro L (2017) β‐Adrenergic modulation of cancer cell proliferation: available evidence and clinical perspectives. J Cancer Res Clin Oncol 143, 275–291. 27709364 10.1007/s00432-016-2278-1 PMC11819197

[mol212657-bib-0009] Cole SW , Nagaraja AS , Lutgendorf SK , Green PA and Sood AK (2015) Sympathetic nervous system regulation of the tumor microenvironment. Nat Rev Cancer 15, 563–572. 26299593 10.1038/nrc3978 PMC4828959

[mol212657-bib-0010] Cole SW and Sood AK (2012) Molecular pathways: β‐Adrenergic signaling in cancer. Clin Cancer Res 18, 1201–1206. 22186256 10.1158/1078-0432.CCR-11-0641 PMC3294063

[mol212657-bib-0011] Curran S , Dundas SR , Buxton J , Leeman MF , Ramsay R and Murray GI (2004) Matrix metalloproteinase/tissue inhibitors of matrix metalloproteinase phenotype identifies poor prognosis colorectal cancer. Clin Cancer Res 10, 8229–8234. 15623598 10.1158/1078-0432.CCR-04-0424

[mol212657-bib-0012] Ebert MS and Sharp PA (2010) MicroRNA sponges: progress and possibilities. RNA 16, 2043–2050. 20855538 10.1261/rna.2414110 PMC2957044

[mol212657-bib-0013] Esteller M (2011) Non‐coding RNAs in human disease. Nat Rev Genet 12, 861–874. 22094949 10.1038/nrg3074

[mol212657-bib-0014] Faulkner S , Jobling P , March B , Jiang CC and Hondermarck H (2019) Tumor neurobiology and the war of nerves in cancer. Cancer Discov 9, 702–710. 30944117 10.1158/2159-8290.CD-18-1398

[mol212657-bib-0015] Fukuyama R , Niculaita R , Ng KP , Obusez E , Sanchez J , Kalady M , Aung PP , Casey G and Sizemore N (2008) Mutated in colorectal cancer, a putative tumor suppressor for serrated colorectal cancer, selectively represses beta‐catenin‐dependent transcription. Oncogene 27, 6044–6055. 18591935 10.1038/onc.2008.204 PMC2574581

[mol212657-bib-0016] Gao Y , Yu H , Liu Y , Liu X , Zheng J , Ma J , Gong W , Chen J , Zhao L , Tian Y *et al* (2018) Long non‐coding RNA HOXA‐AS2 regulates malignant glioma behaviors and vasculogenic mimicry formation via the MiR‐373/EGFR axis. Cell Physiol Biochem 45, 131–147. 29310118 10.1159/000486253

[mol212657-bib-0017] Hou N , Zhang X , Zhao L , Zhao X , Li Z , Song T and Huang C (2013) A novel chronic stress‐induced shift in the Th1 to Th2 response promotes colon cancer growth. Biochem Biophys Res Commun 439, 471–476. 24036270 10.1016/j.bbrc.2013.08.101

[mol212657-bib-0018] Huh JW , Kim HR and Kim YJ (2010) Prognostic value of perineural invasion in patients with stage II colorectal cancer. Ann Surg Oncol 17, 2066–2072. 20182809 10.1245/s10434-010-0982-7

[mol212657-bib-0019] Jing SY , Jing SQ , Liu LL , Xu LF , Zhang F and Gao JL (2017) Down‐expression of miR‐373 predicts poor prognosis of glioma and could be a potential therapeutic target. Eur Rev Med Pharmacol Sci 21, 2421–2425. 28617546

[mol212657-bib-0020] Jobling P , Pundavela J , Oliveria SM , Roselli S , Walker MM and Hondermarck H (2015) Nerve‐cancer cell cross‐talk: a novel promoter of tumor progression. Cancer Res 75, 1777–1781. 25795709 10.1158/0008-5472.CAN-14-3180

[mol212657-bib-0021] Kai AK , Chan LK , Lo RC , Lee JM , Wong CC , Wong JC and Ng IO (2016) Down‐regulation of TIMP2 by HIF‐1alpha/miR‐210/HIF‐3alpha regulatory feedback circuit enhances cancer metastasis in hepatocellular carcinoma. Hepatology 64, 473–487. 27018975 10.1002/hep.28577 PMC5074303

[mol212657-bib-0022] Kohler EM , Chandra SH , Behrens J and Schneikert J (2009) Beta‐catenin degradation mediated by the CID domain of APC provides a model for the selection of APC mutations in colorectal, desmoid and duodenal tumours. Hum Mol Genet 18, 213–226. 18854359 10.1093/hmg/ddn338

[mol212657-bib-0023] Lesko AC , Goss KH and Prosperi JR (2014) Exploiting APC function as a novel cancer therapy. Curr Drug Targets 15, 90–102. 24200292 10.2174/1389450114666131108155418

[mol212657-bib-0024] Li J , Hou N , Faried A , Tsutsumi S and Kuwano H (2010) Inhibition of autophagy augments 5‐fluorouracil chemotherapy in human colon cancer in vitro and in vivo model. Eur J Cancer 46, 1900–1909. 20231086 10.1016/j.ejca.2010.02.021

[mol212657-bib-0025] Liebl F , Demir IE , Rosenberg R , Boldis A , Yildiz E , Kujundzic K , Kehl T , Dischl D , Schuster T , Maak M *et al* (2013) The severity of neural invasion is associated with shortened survival in colon cancer. Clin Cancer Res 19, 50–61. 23147996 10.1158/1078-0432.CCR-12-2392

[mol212657-bib-0026] Liu W , Li M , Chen X , Zhang D , Wei L , Zhang Z , Wang S , Meng L , Zhu S and Li B (2015) MicroRNA‐373 promotes migration and invasion in human esophageal squamous cell carcinoma by inhibiting TIMP3 expression. Am J Cancer Res 6, 1–14. 27073718 PMC4759392

[mol212657-bib-0027] Magnon C (2015) Role of the autonomic nervous system in tumorigenesis and metastasis. Mol Cell Oncol 2, e975643. 27308436 10.4161/23723556.2014.975643 PMC4904882

[mol212657-bib-0028] Magnon C , Hall SJ , Lin J , Xue X , Gerber L , Freedland SJ and Frenette PS (2013) Autonomic nerve development contributes to prostate cancer progression. Science 341, 1236361. 23846904 10.1126/science.1236361

[mol212657-bib-0029] Mauffrey P , Tchitchek N , Barroca V , Bemelmans A‐P , Firlej V , Allory Y , Roméo P‐H and Magnon C (2019) Progenitors from the central nervous system drive neurogenesis in cancer. Nature 569, 672–678. 31092925 10.1038/s41586-019-1219-y

[mol212657-bib-0030] Peng S , Yang X , Liu GJ , Zhang XQ , Wang GL and Sun HY (2015) From the camp pathway to search the ketamine‐related learning and memory. Eur Rev Med Pharmacol Sci 19, 161–164. 25635990

[mol212657-bib-0031] Quaegebeur A , Lange C and Carmeliet P (2011) The neurovascular link in health and disease: molecular mechanisms and therapeutic implications. Neuron 71, 406–424. 21835339 10.1016/j.neuron.2011.07.013

[mol212657-bib-0032] Reiche EM , Nunes SO and Morimoto HK (2004) Stress, depression, the immune system, and cancer. Lancet Oncol 5, 617–625. 15465465 10.1016/S1470-2045(04)01597-9

[mol212657-bib-0033] Roose J , Huls G , van Beest M , Moerer P , van der Horn K , Goldschmeding R , Logtenberg T and Clevers H (1999) Synergy between tumor suppressor APC and the beta‐catenin‐Tcf4 target Tcf1. Science 285, 1923–1926. 10489374 10.1126/science.285.5435.1923

[mol212657-bib-0034] Stetler‐Stevenson WG (2008) The tumor microenvironment: regulation by MMP‐independent effects of tissue inhibitor of metalloproteinases‐2. Cancer Metastasis Rev 27, 57–66. 18058195 10.1007/s10555-007-9105-8 PMC2254553

[mol212657-bib-0035] Tan X , Wang S , Zhu L , Wu C , Yin B , Zhao J , Yuan J , Qiang B and Peng X (2012) cAMP responsive element‐binding protein promotes gliomegenesis by modulating the expression of oncogenic microRNA‐23a. Proc Natl Acad Sci USA 109, 15805–15810. 23019365 10.1073/pnas.1207787109 PMC3465427

[mol212657-bib-0036] Tang J , Li Z , Lu L and Cho CH (2013) β‐Adrenergic system, a backstage manipulator regulating tumour progression and drug target in cancer therapy. Semin Cancer Biol 23, 533–542. 24012659 10.1016/j.semcancer.2013.08.009

[mol212657-bib-0037] Thaker PH , Han LY , Kamat AA , Arevalo JM , Takahashi R , Lu C , Jennings NB , Armaiz‐Pena G , Bankson JA , Ravoori M *et al* (2006) Chronic stress promotes tumor growth and angiogenesis in a mouse model of ovarian carcinoma. Nat Med 12, 939–944. 16862152 10.1038/nm1447

[mol212657-bib-0038] Voorhoeve PM , le Sage C , Schrier M , Gillis AJ , Stoop H , Nagel R , Liu YP , van Duijse J , Drost J , Griekspoor A *et al* (2006) A genetic screen implicates miRNA‐372 and miRNA‐373 as oncogenes in testicular germ cell tumors. Cell 124, 1169–1181. 16564011 10.1016/j.cell.2006.02.037

[mol212657-bib-0039] Wei F , Cao C , Xu X and Wang J (2015) Diverse functions of miR‐373 in cancer. J Transl Med 13, 162. 25990556 10.1186/s12967-015-0523-z PMC4490662

[mol212657-bib-0040] Weng J , Zhang H , Wang C , Liang J , Chen G , Li W , Tang H and Hou J (2017) miR‐373‐3p targets DKK1 to promote EMT‐induced metastasis via the Wnt/beta‐catenin pathway in tongue squamous cell carcinoma. Biomed Res Int 2017, 6010926. 28337453 10.1155/2017/6010926 PMC5350393

[mol212657-bib-0041] Xia T , Chu S , Cui Y , Xu F , Liu Y , Song J , Qian Y , Shao X , Li X , Gu X *et al* (2017) The role of NR2B‐CREB‐miR212/132‐CRTC1‐CREB signal network in pain regulation in vitro and in vivo. Anesth Analg 124, 2045–2053. 28244951 10.1213/ANE.0000000000001880

[mol212657-bib-0042] Xia Y and Wu S (2015) Tissue inhibitor of metalloproteinases 2 inhibits activation of the β–catenin signaling in melanoma cells. Cell Cycle 14, 1666–1674. 25839957 10.1080/15384101.2015.1030557 PMC4614343

[mol212657-bib-0043] Yang JH , Lee EO , Kim SE , Suh YH and Chong YH (2012) Norepinephrine differentially modulates the innate inflammatory response provoked by amyloid‐beta peptide via action at beta‐adrenoceptors and activation of cAMP/PKA pathway in human THP‐1 macrophages. Exp Neurol 236, 199–206. 22609331 10.1016/j.expneurol.2012.05.008

[mol212657-bib-0044] Yaniv SP , Ben‐Shachar D and Klein E (2008) Norepinephrine‐glucocorticoids interaction does not annul the opposite effects of the individual treatments on cellular plasticity in neuroblastoma cells. Eur J Pharmacol 596, 14–24. 18762182 10.1016/j.ejphar.2008.08.006

[mol212657-bib-0045] Zhang Y , Yang J , Cui X , Chen Y , Zhu VF , Hagan JP , Wang H , Yu X , Hodges SE , Fang J *et al* (2013) A novel epigenetic CREB‐miR‐373 axis mediates ZIP4‐induced pancreatic cancer growth. EMBO Mol Med 5, 1322–1334. 23857777 10.1002/emmm.201302507 PMC3799489

