# Supplementary material for: Norepinephrine‐CREB1‐miR‐373 axis promotes progression of colon cancer
Source: Mol Oncol. 2020 Mar 13;14(5):1059–73. doi: 10.1002/1878-0261.12657 (PMC7191185; doi:10.1002/1878-0261.12657)
Supplement: Supplementary file 1 — Fig. S1. Viability changes of HCT116 and RKO induced by NE. Fig. S2. Quantification results of Fig. 2. Fig. S3. Changes of miR‐373‐3p induced by CREB1 overexpression and siCREB1. Fig. S4. Changes of miR‐373‐3p induced by miR‐373 overexpression and miR‐373 inhibitor. Fig. S5. miR‐373 promotes proliferation and metastasis in colon cancer. Fig. S6. Changes of body weight and xenograft volume in experiments of Figure 4E. Fig. S7. Putative miR‐373‐binding sites exists in the 3’‐UTR of TIMP2 and APC, representative images of APC in HCT116 cells transfected with miR‐373 or miR‐Ctrl, and changes of TIMP2 by TIMP2 overexpression. [file MOL2-14-1059-s001.doc]

Supplementary materials

**Norepinephrine-CREB1-miR-373 axis promotes progression of colon cancer**

**
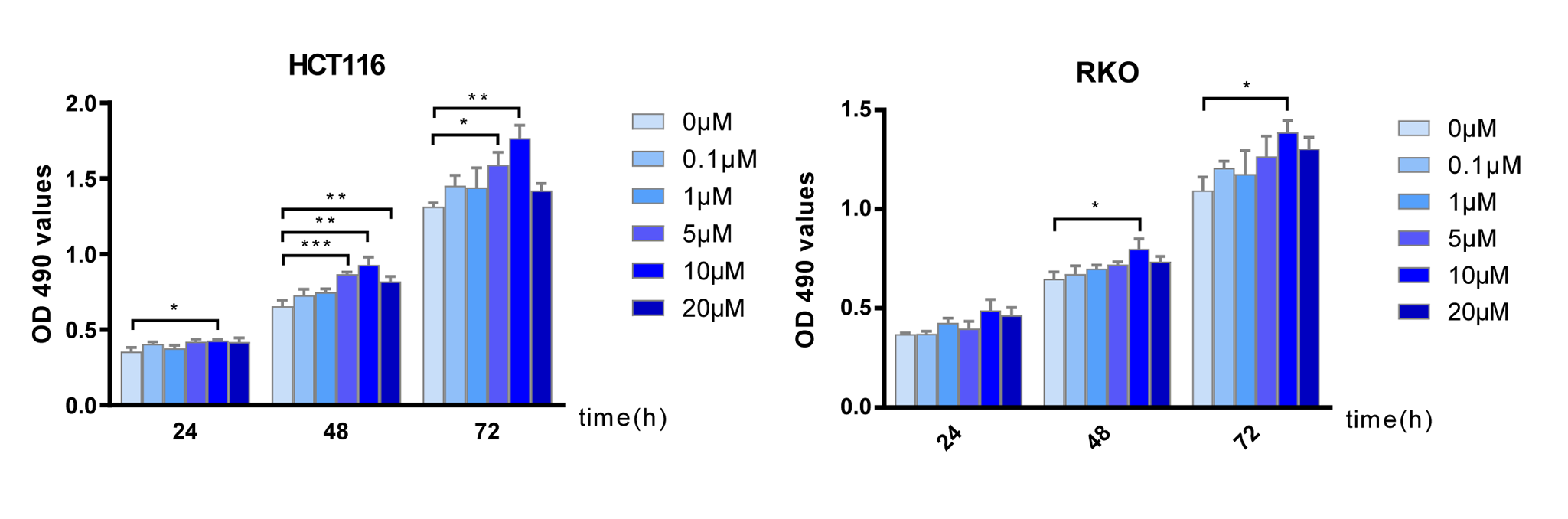
**

**Figure S1.** HCT116 and RKO were treated with different concentrations of NE for 24, 48 and 72h. Changes of cell viability were detected by MTT assay. Error bars are represented as mean ± SD (n=5). P-values were calculated using Student's t-test. * represents P<0.05, ** represents P<0.01, and *** represents P<0.001.


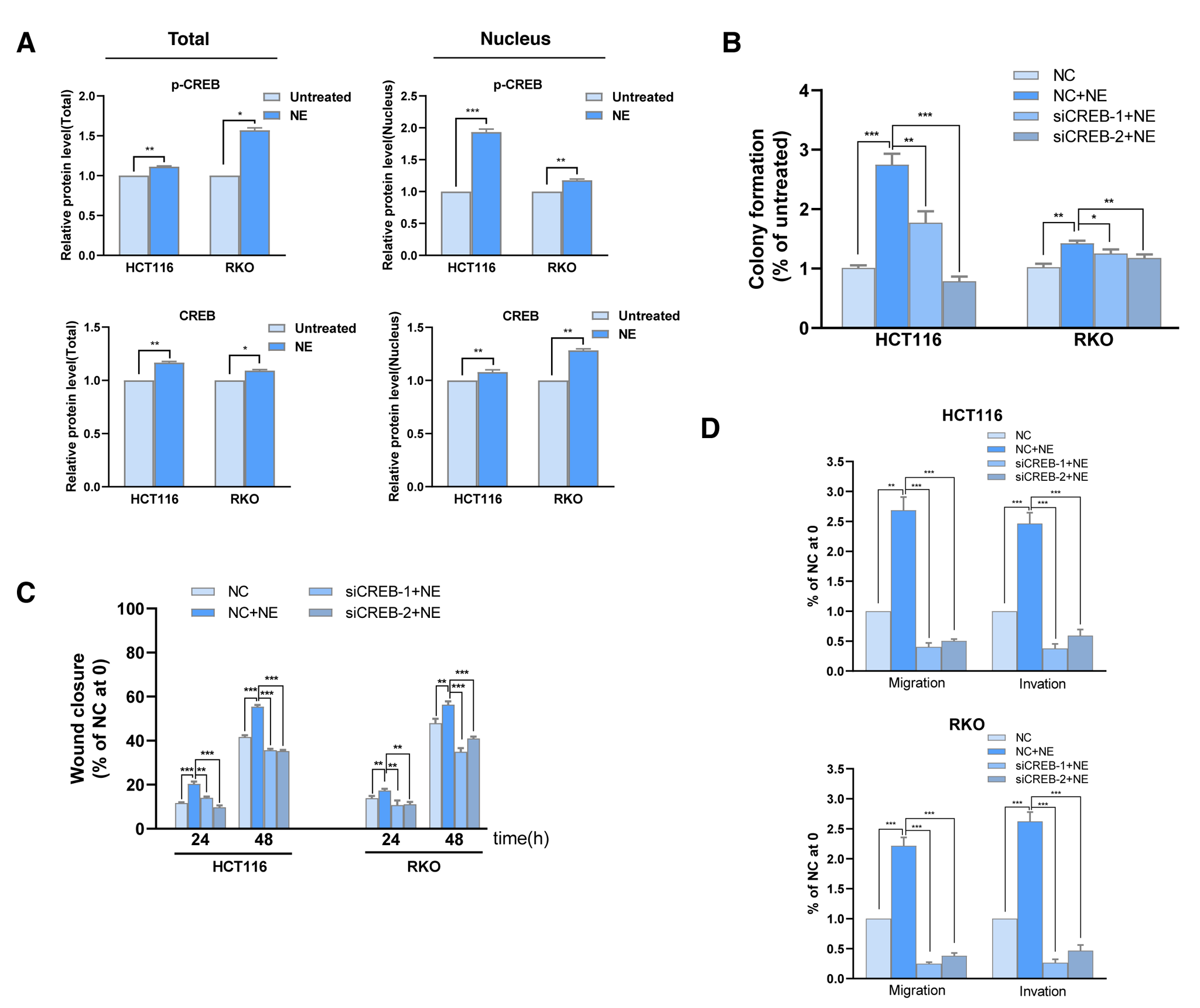


**Figure S2.** A. HCT116 and RKO cells were treated as in Fig 2A. Quantification was performed using Image J soft and normalized. B. HCT116 and RKO cells were treated as in Fig 2D. The percentage of colony formation assay has been indicated. C. HCT116 and RKO cells were treated as in Fig 2E. The percentage of wound closure has also been indicated. D. HCT116 and RKO cells were treated as in Fig 2F. The percentages of migration and invasion have also been indicated. Error bars are represented as mean ± SD (n=5). P-values were calculated using Student's t-test. * represents P<0.05, ** represents P<0.01, and *** represents P<0.001.

**
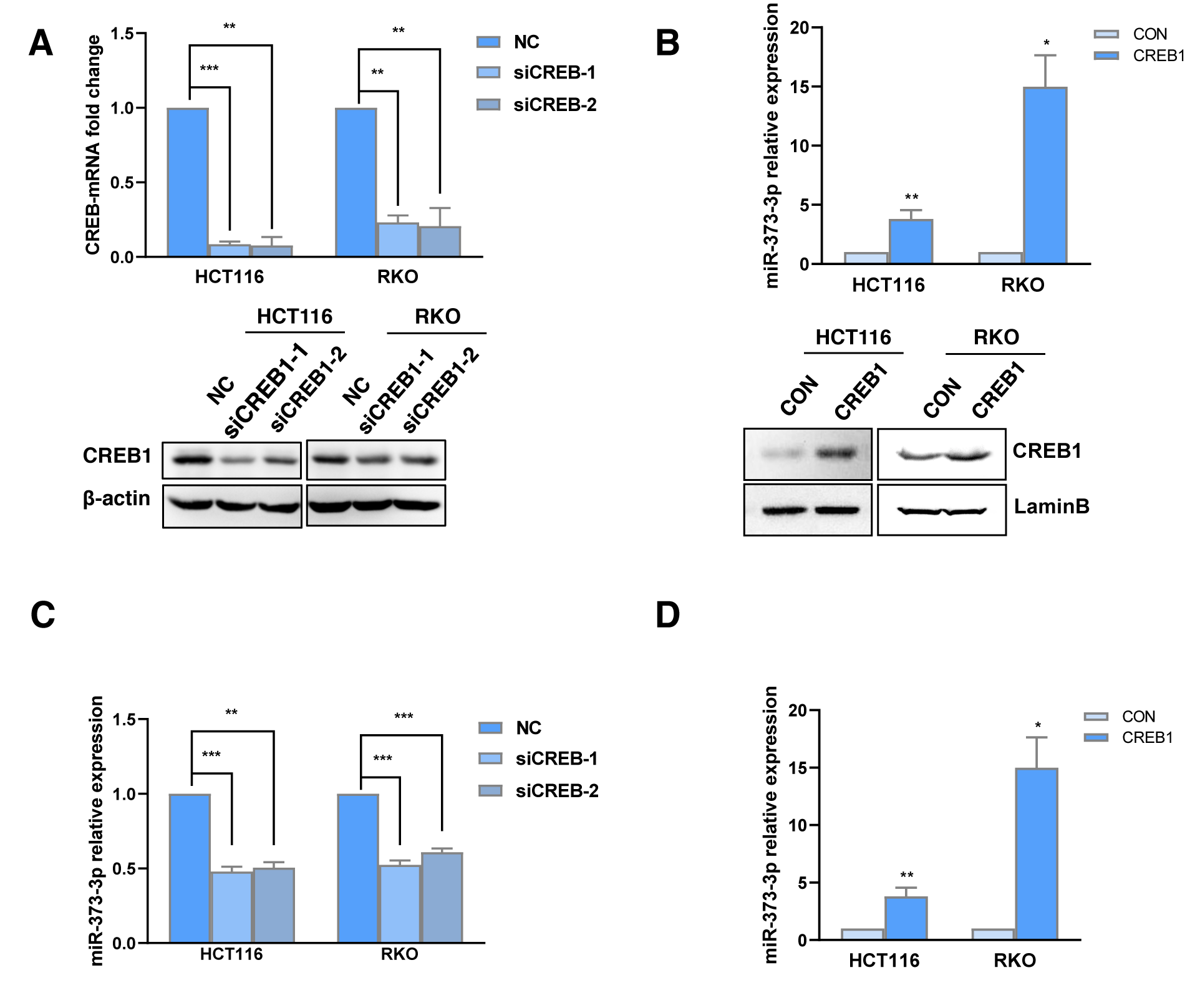
**

**Figure S3.** A. HCT116 and RKO were transfected with siCREB1-1, siCREB1-2, or NC. B. HCT116 and RKO were transfected with CREB1 or CON. QRT-PCR and western blotting detected changes of CREB. C. HCT116 and RKO were transfected with siCREB1-1, siCREB1-2, or NC. D. HCT116 and RKO were transfected with CREB1 or CON. Changes of miR-373-3p were detected using qRT-PCR. Error bars are represented as mean ± SD (n=5). P-values were calculated using Student's t-test. * represents P<0.05, ** represents P<0.01, and *** represents P<0.001.

**
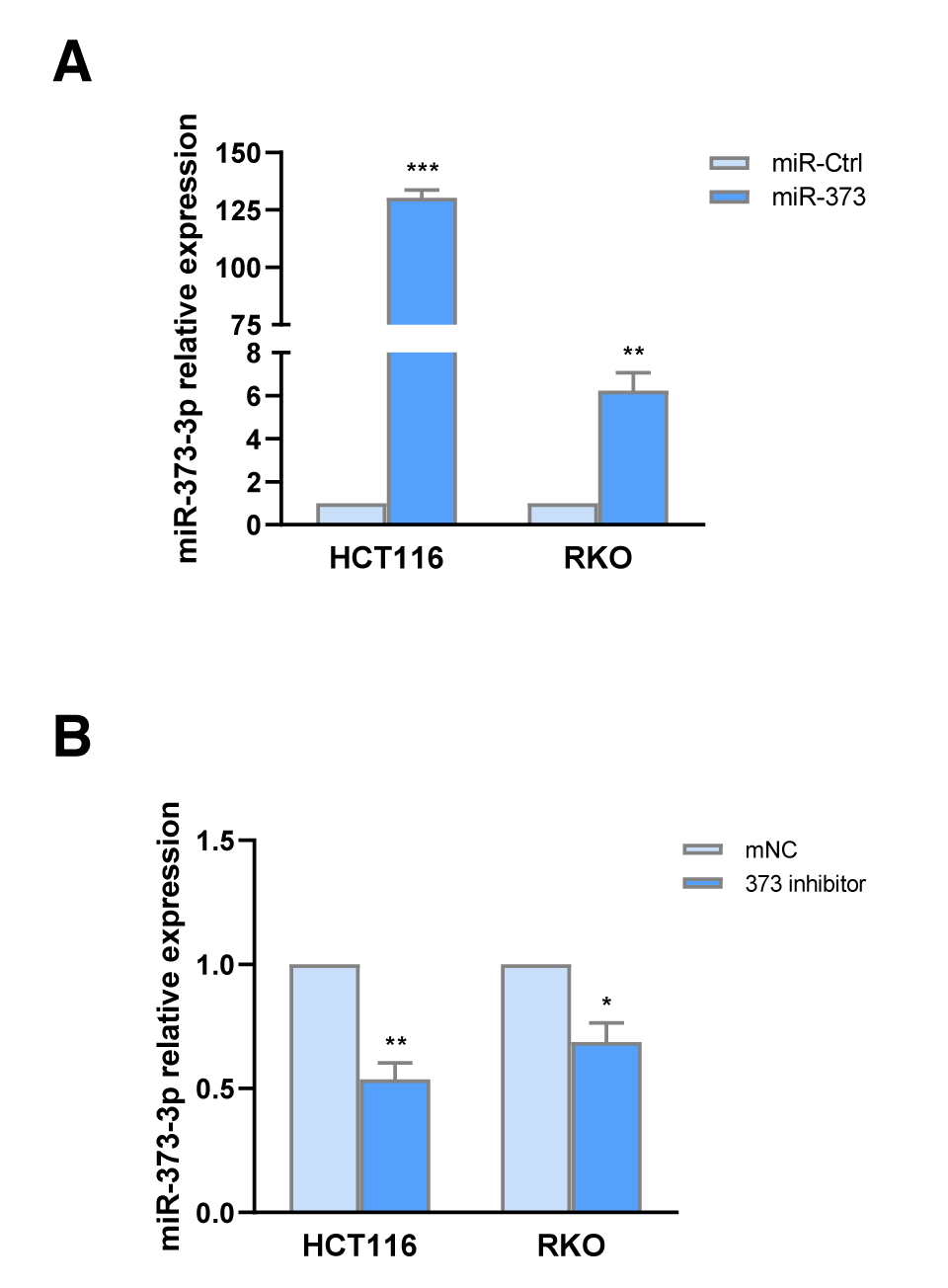
**

**Figure S4.** A. HCT116 and RKO were transfected with miR-373 or miR-Ctrl. B. HCT116 and RKO were transfected with miR-373 inhibitor (373 inhibitor) or mNC. Changes of miR-373-3p were detected using qRT-PCR. Error bars are represented as mean ± SD (n=5). P-values were calculated using Student's t-test. * represents P<0.05, ** represents P<0.01, and *** represents P<0.001.


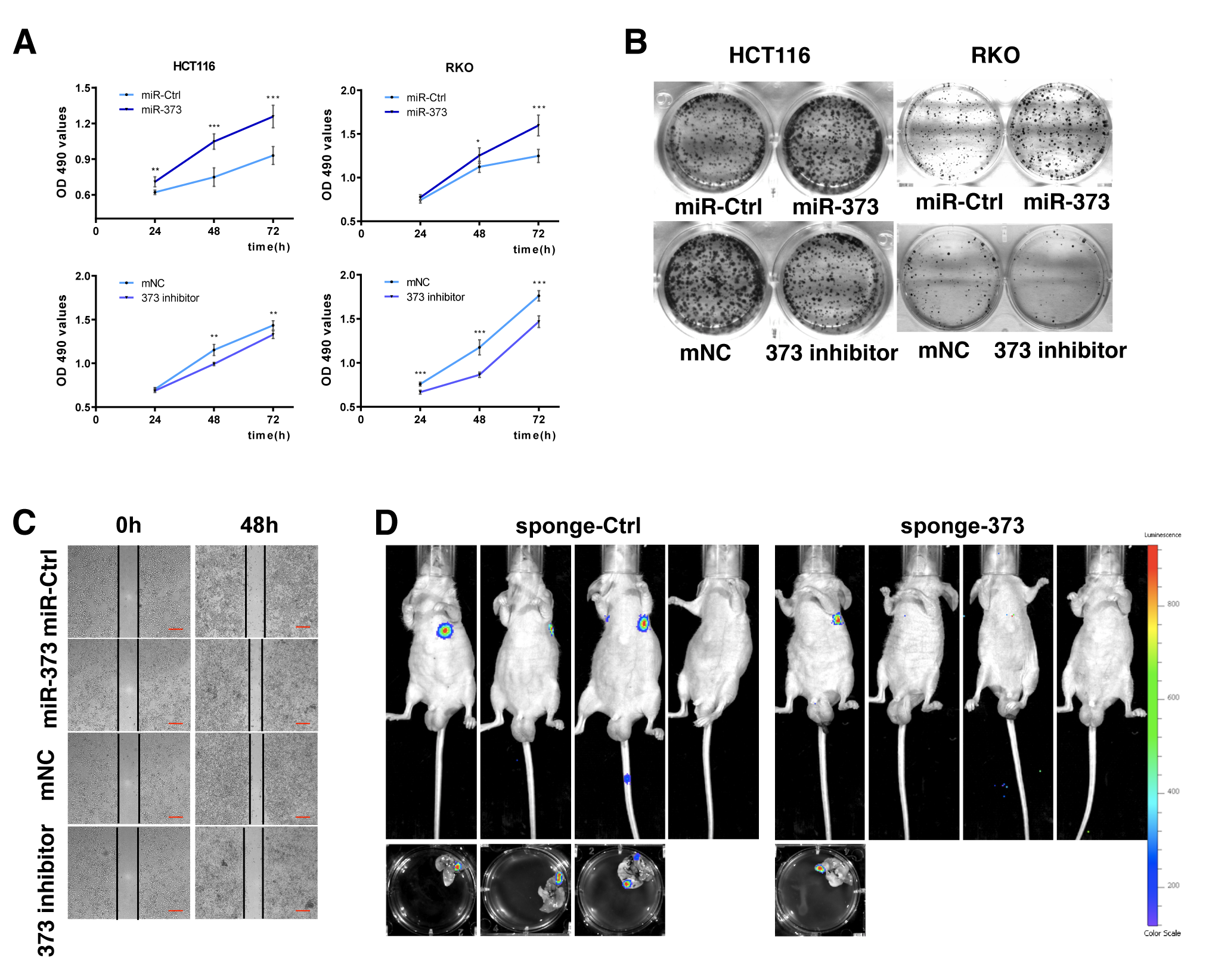


**Figure S5.** miR-373 promotes proliferation and metastasis in colon cancer.

HCT116 and RKO were transfected with miR-373 or miR-Ctrl, or miR-373 inhibitor (373 inhibitor), or mNC. MTT assay (A) and colony formation assay (B) were performed. C, Wound healing assay was performed in RKO cells transfected with miR-373 or miR-Ctrl, or miR-373 inhibitor (373 inhibitor), or mNC. Scale bars represent 100 μm. D, Nude mice were injected HCT116/sponge-miR-Ctrl or HCT116/sponge-miR-373 cells (2×10^6^) in the tail vein. At day 40, they were imaged for bioluminescence. The organs with bioluminescence signal of the same animal were removed out for idiographic image. Dots colors on the right correspond to luminescence counts as per the scale. Error bars are represented as mean ± SD (n=5). P-values were calculated using Student's t-test. * represents P<0.05, ** represents P<0.01, and *** represents P<0.001.

**
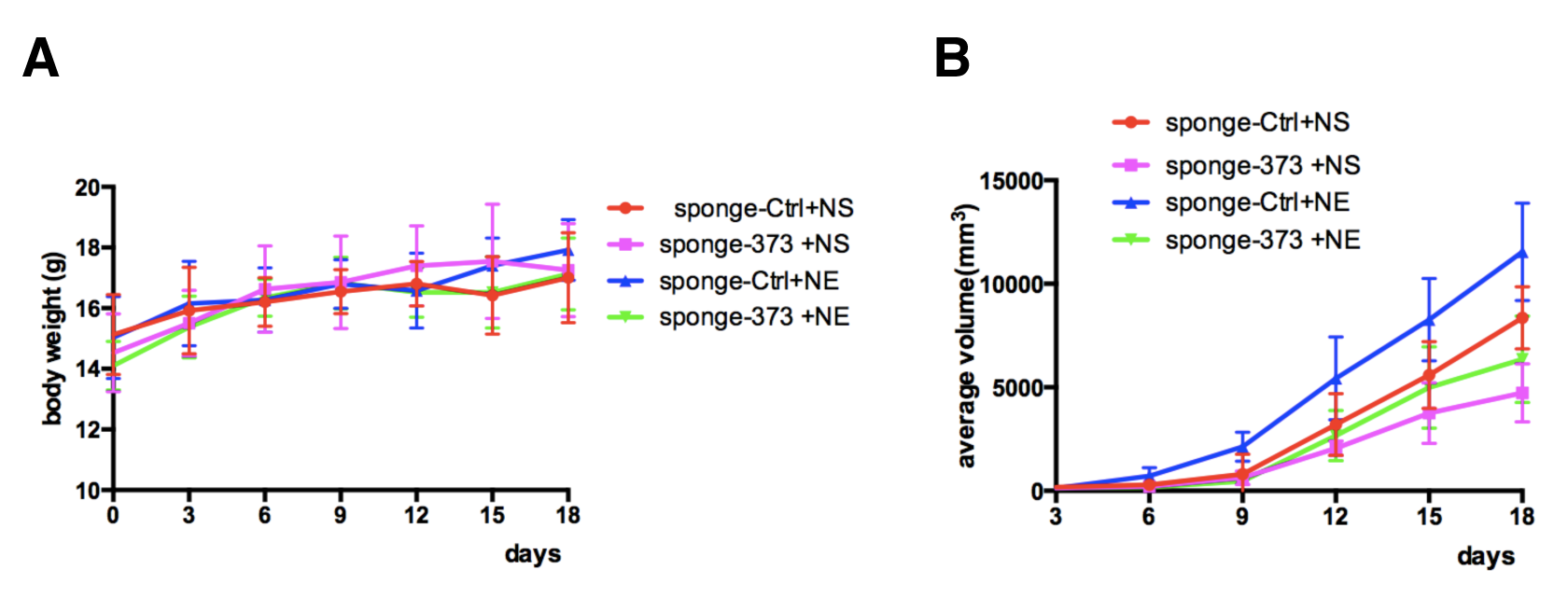
**

**Figure S6.** HCT116/sponge-miR-Ctrl or HCT116/sponge-miR-373 cells (3×10^6^) were subcutaneously injected into the posterior flanks of nude mice separately, with NE or saline (NS) intraperitoneal injected everyday. Body weight (A) and xenograft volume (B) were measured. Error bars are represented as mean ± SD (n=4-5).


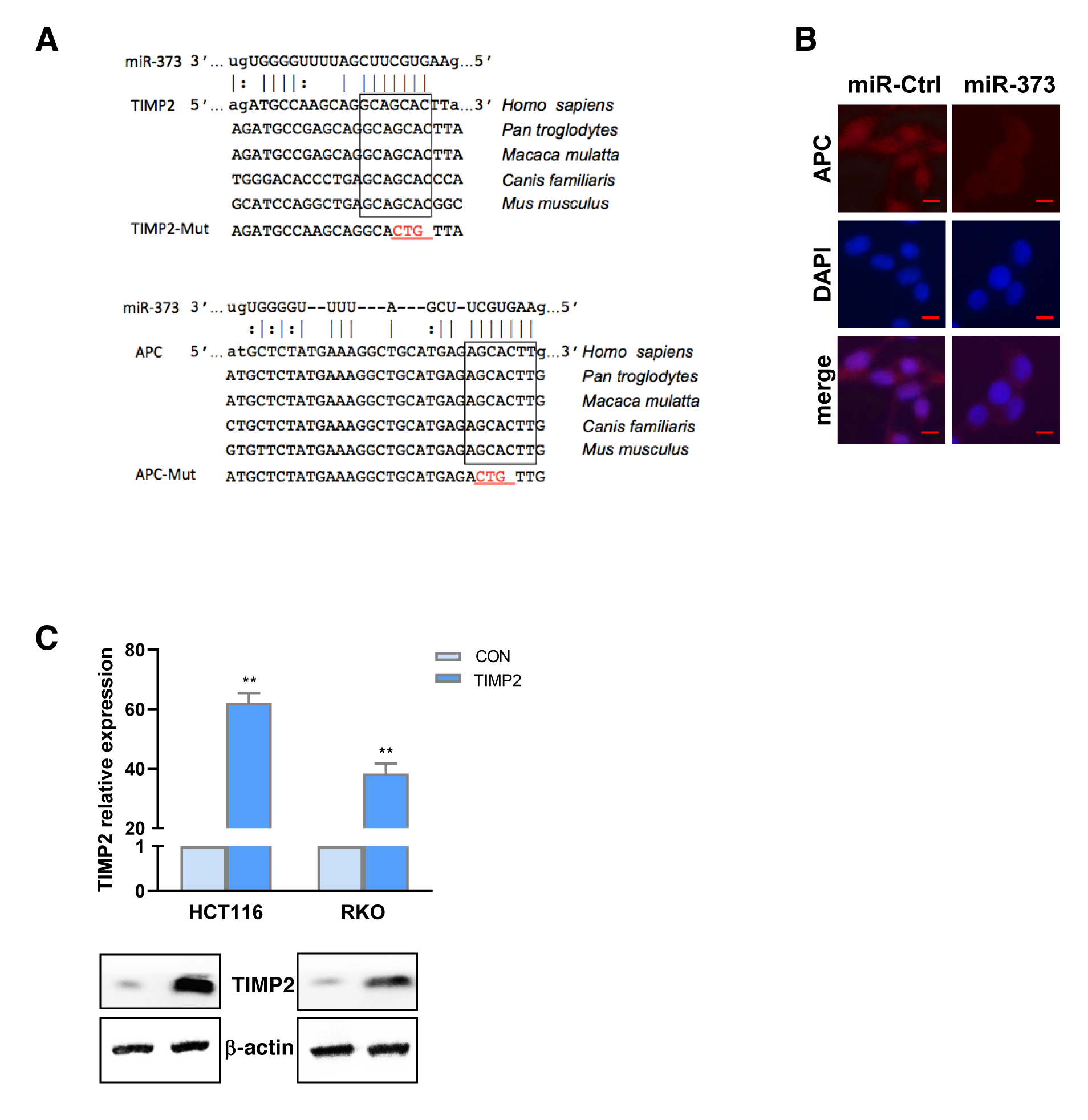


**Figure S7.** A. Putative miR-373-binding sites exists in the 3’-UTR of TIMP2 and APC, are conserved in many species. Their three point mutations in binding sites were generated as indicated in red. These target sequences and their mutated ones were subcloned into pmirGLO vector. B. Representative images of APC in HCT116 cells transfected with miR-373 or miR-Ctrl, with APC stained in red and DAPI in blue. Scale bars represent 10 μm. C. HCT116 and RKO were transfected with TIMP2 or CON106. qRT-PCR and western blotting detected changes of TIMP2. Error bars are represented as mean ± SD (n=5). P-values were calculated using Student's t-test. ** represents P<0.01, and *** represents P<0.001.
