# Supplementary material for: Norepinephrine‐CREB1‐miR‐373 axis promotes progression of colon cancer
Source: Mol Oncol. 2020 Mar 13;14(5):1059–73. doi: 10.1002/1878-0261.12657 (PMC7191185; doi:10.1002/1878-0261.12657)
Supplement: Supplementary file 2 — Table S1. Sequences of interference oligonucleotides. Table S2. Primers for qRT‐PCR. Table S3. Primer pairs used in ChIP assay. Table S4. Insert sequence of pGL3‐promoter plasmids. Table S5. Insert sequences of pmirGLO plasmids. [file MOL2-14-1059-s002.pdf]

Supplementary tables

Norepinephrine-CREB1-miR-373 axis promotes colon cancer growth

Table S1. Sequences of interference oligonucleotides.

Name	Sequence
NC	sense: UUCUCCGAACGUGUCACGUTT<br>antisense: ACGUGACACGUUCGGAGAATT
siCREB1-1	sense: GCAGCUCGAGAGUGUCGUATT<br>antisense: UACGACACUCUCGAGCUGCTT
siCREB1-2	sense: GCGAAGGGAAAUUCUUUCATT<br>antisense: UGAAAGAAUUUCCCUUCGCTT
mNC	CAGUACUUUUGUGUAGUACAA
miR-373 inhibitor	ACACCCCAAAAUCGAAGCACUUC

Table S2. Primers for qRT-PCR.

Primer name	Sequence
miR-373-3P RT	GTCGTATCCAGTGCGTGTCGTGGAGTCGG<br>CAATTGCACTGGATACGACACACCCCAA
miR-373-3P F	ATCCAGTGCGTGTCGTGG
miR-373-3P R	AGCCTGAAGTGCTTCGATT
u6 RT	CGCTTCACGAATTTGCGTGTCAT
u6 F	GCTTCGGCAGCACATATACTAAAAT
u6 R	CGCTTCACGAATTTGCGTGTCAT
CREB1-F	TTCTCCGGAACACAGATTTCA
CREB1-R	TGTCCATCAGTGGTCTGTGC
β-actin F	CCAACCGCGAGAAGATGA
β-actin R	CCAGAGGCGTACAGGGATAG
TIMP2-F	TAGTGATCAGGGCCAAAGCG
TIMP2-R	AGGGCACGATGAAGTCACAG
APC-F	CTCGGAAATGGGGTCCAAGG
APC-R	GGAGGGACATTTTTGACCGC

Table S3. primer pairs used in ChIP assay.

primer pair	sequence
primer pair 1-F	GTTGAGTGGATGACTGGTGGAA
Primer pair 1-R	ACCCTGCTCCCTTTGATCCTA
primer pair 2-F	AGCTGCAGTGAACCTTGTTCTT
primer pair 2-R	CAATACTCACAGAGGCCAATGA
primer pair 3-F	AGTGGAAAGTGCTGCGACAT
primer pair 3-R	TTTCCGGAGCCATTACAGCC
primer pair 4-F	CCAAGGGGCTGTATGCACAA

primer pair 4-R	GGTTGGCCTCCAATCATCCA

Table S4. Insert sequence of pGL3-promoter plasmids

	Plasmid name	Sequence
primer pair 2 region	pGL-2-wt	AGACCTGA*GGTGTGGACGTATCATTGGCCTCTGTGAG*<br>*TATTG*CTATGTTATTTTATTTTCTTTCATTTTACTTTATTTT<br>TTAGGGTCAGGATCTCACTGTCGCC*CAGGATGAAGTG*<br>CACAGGTAG*GATGATGGCGCC*TTGCAGCCTCGACCTC<br>GGGACTCACCCTTTTAGCCTCCAGT
	pGL-2-mt	AGACCTGA*GGTGTGGcacTATCATTGGCCTCTGTGAGT*<br>*ATTG*CTATGTTATTTTATTTTCTTTCATTTTACTTTATTTTT<br>TAGGGTCAGGATCTCACTGTCGCC*CAGGAggtAGac*CA<br>CAGGTAG*GAccgTGGCGCC*TTGCAGCCTCGACCTCCT<br>GGGACTCACCCTTTTAGCCTCCAGT
primer pair 3 region	pGL-3-wt	GTGGAAAGTGCTGCGACATTTGAGCGTCACCGGTGAC<br>GCCCATATCAACGGATGCCGTGGAGCTCGGTCTTCTG<br>CAGG
	pGL-3-mt	GTGGAAAGTGCTGCGACATTTGAGtgatCCGGtctaCCCA<br>TATCAACGGATGCCGTGGAGCTCGGTCTTCTGCAGG

Table S5. Insert sequences of pmirGLO plasmids.

Plasmid name	Sequence
APC-WT	CTCATCCCTTCCTCGAGTAAGCACTTGGACTGTACAATAGAAGCAGA<br>ATTAGATGCTCAGCACTTAGCTAGTCAGAGTGAAGTATAATCAGCACT<br>TTAGCAGATCGCAAGCCCTAGGAAAAGCAGCGCAGATAGCACTTCAT<br>GACTCGTGATGCTCTATGAAAGGCTGCATGAGAGCACTTGTCTAGAT<br>CGAGTACCTAAAAATAAAGCACCTAC
APC-MT	CTCATCCCTTCCTCGAGTAACTGTTGGACTGTACAATAGAAGCAGAA<br>TTAGATGCTCACTGTTAGCTAGTCAGAGTGAAGTATAATCACTGTTTA<br>GCAGATCGCAAGCCCTAGGAAAAGCAGCGCAGATACTGTTCATGAC<br>TCGTGATGCTCTATGAAAGGCTGCATGAGACTGTTGTCTAGATCGAG<br>TACCTAAAAAAACTGCCTAC
TIMP2-WT	CCGTCACAGATGCCAAGCAGGCAGCACTTAACGAGATCGCTTTCTG<br>CATCGTGGAAGCATTTGC
TIMP2-MT	CCGTCACAGATGCCAAGCAGGCACTGTTAACGAGATCGCTTTCTGC<br>ATCGTGATTCATTTGC
